# Blood-based biomarker discovery for early pregnancy loss using integrative multi-omics strategies

**DOI:** 10.1016/j.ebiom.2026.106253

**Published:** 2026-04-13

**Authors:** Yue Shi, Yongkang Yang, Xianghao Guo, Shuai Shi, Qin Li, Chi Chiu Wang, Liona C. Poon, Pui Wah Chung, Jianan Xia, Yongfei Wang, Xingqiang Lai, Yueqiong Ni, Xiaoyan Chen, Yao Wang

**Affiliations:** a Department of Obstetrics and Gynaecology, Faculty of Medicine, Prince of Wales Hospital, The Chinese University of Hong Kong, Hong Kong SAR; b The Second Clinical Medical College, Shaanxi University of Chinese Medicine, Xianyang, China; c Department of Obstetrics and Gynecology, The Second Affiliated Hospital of Shaanxi University of Chinese Medicine, Xianyang, China; d Li Ka Shing Institute of Health Sciences, Chinese University of Hong Kong, Hong Kong SAR; e School of Medicine, Warshel Institute for Computational Biology, The Chinese University of Hong Kong, Shenzhen, China; f Department of Obstetrics and Gynecology, Maternal-Fetal Medicine Institute, Shenzhen Baoan Women's and Children's Hospital, Shenzhen, China; g State Key Laboratory of Metabolic Dysregulation and Prevention and Treatment of Esophageal Cancer, Shanghai Key Laboratory of Diabetes Mellitus, Department of Endocrinology and Metabolism, Shanghai Diabetes Institute, Shanghai Clinical Center for Diabetes, Shanghai Sixth People's Hospital Affiliated to Shanghai Jiao Tong University School of Medicine, Shanghai, China

**Keywords:** Early pregnancy loss, Miscarriage, Multi-omics, Proteomics, Metabolomics, Integrative analysis, ANGPTL4, PD-L1

## Abstract

**Background:**

Early pregnancy loss (EPL), a spontaneous death of the embryo or foetus occurring within the first trimester, is a major challenge for human reproduction with profound adverse consequences for women's health. Currently, reliable blood-based biomarkers for EPL remain limited. Therefore, there is an urgent need to discover novel biomarkers for EPL using a multi-omics-based approach to facilitate early detection and timely management.

**Methods:**

In the discovery cohort, 40 patients with EPL and 40 healthy pregnancies (HP) at 7–13 weeks of gestation were enrolled. Serum proteins and metabolites were assayed by Olink® technology and ultra-performance liquid chromatography coupled to tandem mass spectrometry (UPLC-MS/MS), respectively. Biomarkers were defined by false discovery rate (FDR) < 0.05 and fold change (FC) > 1.2. Random forest (RF) and logistic regression (LR) models incorporating selected biomarkers were employed to develop diagnostic models for EPL. In the external validation cohort, we prospectively enrolled 142 pregnancies at 7–10 gestational weeks, including 47 subjects who subsequently developed EPL and 95 pregnancies with full-term birth. Serum levels of selected biomarkers were quantified by ELISA.

**Findings:**

The combined proteomics and metabolomics screening identified 26 proteins and 21 metabolites significantly changed in the EPL group and tightly associated with EPL-related clinical phenotypes, with functional enrichment in immunoregulation and lipid oxidation processes. Moreover, integrating serum levels of angiopoietin-like 4 (ANGPTL4), programmed death-ligand 1 (PD-L1), neutrophil%, and lymphocyte% achieved an AUC of 0.944 (95% CI: 0.835–1.000) in the random forest model and 0.954 (95% CI: 0.875–1.000) in the logistic regression model to discriminate EPL from HP. Importantly, this four-biomarker model achieved an AUC of 0.857 (95% CI: 0.747–0.968) in the random survival forest model and a C-index of 0.804 (95% CI: 0.685–0.973) in the validation cohort for EPL prediction.

**Interpretation:**

Our integrative omics study reveals a panel of potential circulating biomarkers for EPL, which further offer mechanistic insights into EPL pathogenesis, including impaired maternal immune tolerance and dysregulated lipid metabolism pathways. Moreover, the newly identified biomarkers exhibit promising diagnostic and predictive performance for EPL, underscoring its clinical translational value for human reproduction and maternal–foetal health.

**Funding:**

This study was supported by Research Grants Council (RGC) Germany/Hong Kong Joint Research Scheme (G-CUHK415/25), 1+1+1 CUHK-CUHK(SZ)-GDST Joint Collaboration Fund (2025A0505000077), CUHK HOPE BWCH Collaborative Medical Research Fund (CF2025002), Shenzhen Medical Research Fund (C2501040), and Shenzhen Science and Technology Program (RCYX20210609104608036).

**Research in context**

**Evidence before this study**

Early pregnancy loss (EPL) is a spontaneous embryo death in the first trimester with profound adverse consequences for women. Traditional indices like beta-human chorionic gonadotrophin exhibit unsatisfactory performance for EPL diagnosis and prediction. Moreover, the circulating protein and metabolite profiles in EPL are still under-investigated. Hence, there is a pressing need for blood-based biomarker discovery using advanced multiomics-based strategies, which will enable accurate EPL detection and mechanistic insights.

**Added value of this study**

We applied a multi-omics biomarker screening strategy incorporating Olink Proteomics and untargeted metabolites. A total of 26 proteins and 21 metabolites were profiled in serum samples as potential biomarkers for EPL. Further integrative analysis reveals that EPL is potentially associated with immunology and lipid oxidation dysregulation. Importantly, applying serum levels of angiopoietin-like 4 (ANGPTL4) and programmed death-ligand 1 (PD-L1) with blood lymphocyte proportion achieved an AUC of 0.944 to discriminate EPL from healthy pregnancy. Importantly, this model also resulted in an AUC of 0.857 in our independent, prospective validation cohort for EPL prediction.

**Implications of all the available evidence**

Women with EPL show distinct circulating protein and metabolite profiles compared to healthy pregnancies. We identified a list of potential biomarkers for EPL, which also provide possible targets for further mechanistic study. Applying serum levels of ANGPTL4 and PD-L1 may enhance the assessment of miscarriage risk in clinical practice.

## Introduction

Early pregnancy loss (EPL), also known as miscarriage, is a spontaneous death of the embryo or foetus occurring in the first trimester of gestation.^1^ It represents a serious complication affecting 10%–15% of clinically recognised gestations and threatens human reproduction worldwide.^2^, ^3^, ^4^ EPL renders profound adverse consequences in women, accompanied by haemorrhage, infection, and complications secondary to septic miscarriage.^1^ Importantly, it further impairs subsequent fertility and is associated with an increased risk of obstetric complications in later pregnancies.^5^ Moreover, EPL also poses emotional distress and depressive symptoms in affected women that can persist for years.^5^ Given its severe negative consequences, EPL is a pressing issue that necessitates the urgent development of innovative discriminative biomarkers for the early detection and intervention.

Biomarkers are measurable indicators enabling early prediction, precise diagnosis, and therapeutic treatment. However, clinically robust biomarkers capable of detecting EPL remain elusive. Although decreased blood β-human chorionic gonadotrophin (β-HCG) levels are associated with the occurrence of miscarriage,^6^^,^^7^ its diagnostic and discriminative performance in EPL is suboptimal.^8^ Recent meta-analyses further suggested that conventional clinical parameters, such as progesterone and platelet-to-lymphocyte ratio, are insufficient for EPL diagnosis.^8^^,^^9^ Despite several protein-based biomarkers being identified,^10^^,^^11^ these candidates were largely identified via hypothesis-driven or literature-based screening with potential risk of selection bias. Moreover, metabolite-based biomarkers and metabolism signatures in the context of EPL are under investigation,^12^, ^13^, ^14^ leaving an unknown gap to understand EPL from a metabolic perspective. Therefore, there is a pressing need for a comprehensive, multidimensional approach to biomarker discovery in EPL by integrating both proteomics and metabolomics data.

To accurately capture the molecular signal and screen the biomarker candidates, high-throughput, omics-based approaches are needed. Olink® is a state-of-the-art proteomics technology, enabling simultaneous analysis of over 1000 unique hormones/cytokines from less than 10 μL of serum.^15^ For metabolomics, ultra-performance liquid chromatography coupled to tandem mass spectrometry (UPLC-MS/MS) shows its high selectivity with low-detection limits for detecting small molecular metabolites.^16^ These advanced methodologies have been widely applied in biomarker discovery,^11^ with great clinical translational potential, and offer direct mechanistic insights into functional readouts.^10^

Herein, we organised a nested case–control study of women with EPL and maternal and gestational age-matched healthy pregnancies (HP) at 7–13 weeks of gestation for multi-omics screening to profile metabolite and protein biomarkers. We further conducted integrative analysis to contextualise their putative contributions and interactions to EPL pathophysiology. The screened biomarkers were then validated in an independent prospective cohort with patients who subsequently developed EPL. Our findings identified informative biomarkers and developed a multivariable model with robust discriminative and predictive performance for EPL. It also advances the mechanistic insights into the underlying pathophysiology.

## Methods

### Ethics

The study protocol was approved by the human research ethics committee of the Second Affiliated Hospital of Shaanxi University of Chinese Medicine (IRB Ref.: CYL-SQ2023016) and Department of Obstetrics and Gynaecology, Shenzhen Baoan Women's and Children's Hospital (IRB Ref. LLCS-2025-02-04-07-KS). This study was in accordance with the criteria established by the Declaration of Helsinki. All participants signed informed consent before recruitment and consented independently when donating samples. All data obtained and generated during the study were kept confidential.

### Human participants and study design

The diagnostic criteria of EPL are defined as a nonviable, intrauterine pregnancy with either an empty gestational sac or a gestational sac containing an embryo or foetus without foetal heart activity within the first 12^+6/7^ weeks of gestation.^7^ EPL cases with genetic/chromosomal anomalies, intrauterine/systemic infection, or an acute illness within the past seven days before sample collection were excluded. Healthy control cases had a full-term delivery without major pregnancy-associated complications, such as preeclampsia and preterm birth. The gestational stage was estimated according to the first date of the last menstrual period and ultrasonographic examination.^17^

In the discovery cohort, we enrolled 40 patients with confirmed EPL and 40 HP at the same stage in early pregnancy (7–13 weeks of gestation). Fasting blood samples were collected at the initial prenatal visit in the HP group, and on the date of dilatation and curettage surgery (after the diagnosis of EPL). The two groups were matched for maternal age and gestational weeks. In the validation phase, we identified 47 women who subsequently developed EPL at 7–13 weeks of gestation from our established prenatal cohort. Banked plasma samples collected at 7–10 weeks of gestation (prior to EPL onset) were retrieved for analysis. These cases were matched with 95 healthy controls who delivered at term without major obstetric complications, matched for maternal age and gestational age at sample collection. Approximately 2 mL of fasting peripheral venous blood was collected from each participant and centrifugation at 3000 rpm for 15 min and frozen at −80 °C (Supplementary Figure S1).

### Olink Proteomics detection and analysis in human serum

Antibody-based technology (Olink Proteomics AB, Uppsala, Sweden) (RRID: SCR_003899) was used for proteomics profiling in serum samples via the Olink Target 96 Inflammation (Cat. No.: 95302), Development (Cat. No.: 95352), and Metabolism Panels (Cat. No.: 95340) (Supplementary Table S1). Data from testing were presented as normalised protein expression (NPX) units on a log2 scale. Data quality control, normalisation, and standardisation were performed to minimise both intra- and inter-assay variation using the Olink NPX Manager software (v2.2). Proteins that are not detected in >80% of the samples were excluded from analysis. Missing values of protein (if any) were mean imputed, following previous publication^18^^,^^19^ R package *OlinkAnalyze* (v4.5.0) was employed for data analysis.^20^

### Circulating metabolomics screening and analysis

Targeted metabolomics profiling of serum samples was performed by Metabo-Profile (Shanghai, China) using their Q300 Metabolite Array Kit (Patent: CN109298115B). The detection method has been elaborated in the previous publication.^21^ In brief, 20 μL serum was thawed on ice and transferred to an Eppendorf epMotion Workstation and further quantified by a UPLC-MS/MS system (ACQUITY UPLC-Xevo TQ-S, Waters Corp., Milford, MA, USA). Raw ion intensities were processed using the TMBQ software (v1.0, Metabo-Profile, Shanghai, China) to perform peak integration, calibration, and quantitation. The concentration of targeted metabolites was calculated by MS using the external calibration curve and IS. Metabolites detected in fewer than 80% of samples were excluded from further analyses. Missing metabolite values were imputed, if necessary, by replacing them with one-tenth of the minimum detected value for that metabolite across all samples, as recommended by previous studies.^22^^,^^23^ The detected metabolites in blood samples were listed in Supplementary Table S2.

### Immunoassay of serum levels of ANGPTL4 and sPD-L1

ELISA was employed for targeted protein measurement in human blood samples. Serum ANGPTL4 levels were measured using the ELISA Duoset Development System (Cat. DY3485, R&D, USA). And PD-L1 levels were measured using commercial kits (Cat.DB7H10, R&D, USA). The sensitivity, limit of detection, and inter- and intra-assay coefficient of variation (COV) of these two kits are shown in Reagent Validation. The intra-plate variations were controlled to 10%.

### Differential analysis in biomarker discovery

Principal components analysis (PCA) of the protein and metabolite concentration was performed with R package *FactoMineR* (v2.13)^24^ and *factoextra* (v2.0.0)^25^ primarily to inspect the dataset's structure and to validate the discernibility of sample groups.^26^ The difference of principle components between groups was evaluated by permutation test. The first principal component represents the direction of maximum variance in the data, which *p* value reflects the pattern of variation among samples. Subsequently, we engaged in orthogonal partial least squares-discriminant analysis (OPLS-DA) with R package *ropls* (v1.42.0),^27^ which models variances in predictor variables against the dependent variable, ensuring that both low- and high-abundance analytes exerted comparable influence on the model. The model's effectiveness was evaluated based on R2 (goodness of fit) and Q2 (goodness of prediction) metrics.^27^ The comparisons of protein and metabolite concentration between EPL and HP groups were conducted with two-tailed Student's t-test with the R package *rstatix* (v0.7.3).^28^ The potential impact of multiple covariates, such as maternal age and gestational weeks on protein and metabolite levels was adjusted using multiple linear regression. The Benjamini-Hochberg false discovery rate (FDR) method, with an FDR significance threshold of 0.05, was applied to control for multiple testing. The fold change (FC) was applied to compare the relative change between the EPL group and healthy control.

### Enrichment analysis

To reveal the potential pathways in EPL pathology, Gene Ontology (GO) and Kyoto Encyclopedia of Genes and Genomes (KEGG) enrichment analyses were performed using R package *clusterprofiler* (v4.18.4),^29^ and visualised by R package *enrichplot* (v1.30.5)^30^ and *ggplot2* (v4.0.2).^31^ All significantly DEPs and DEMs were mapped to each term or pathway of the GO or KEGG database, and the GO term or KEGG pathway that was significantly enriched in DEPs compared to the whole genome background was identified using a hypergeometric test. Moreover, the protein–protein interaction (PPI) network of DEPs was constructed using STRING database (v12.5), with the interaction threshold >0.4.

### Correlation analysis

To investigate the clinical implication of the proteins and metabolites concentration and the interaction of the proteins and metabolites, partial correlation analysis was performed by R package *ppcor* (v1.1)^32^ for the correlation of clinical features and DEPs and DEMs, and for building the networks of DEPs and DEMs. Covariates were maternal age, BMI, and gestational weeks. *p*-value with the cutoffs of 0.05 was applied to identify the significant correlations. Heatmaps were constructed with the R package *ComplexHeatmap* (v2.26.1),^33^ and network plots were generated with the R package *gglpot2* (v4.0.2).^31^

### Mediation analysis

Mediation analysis was conducted to further explore the relationships between independent variables (proteins or metabolites) and dependent variables (clinical parameters). R package *mediation* (v4.5.1) was applied to estimate the average causal mediation effect (ACME), direct effect (DE), total effect and the proportion of mediation, where bootstrapping was used to obtain robust confidence intervals for the indirect effects.^34^ *p*-value cutoffs of 0.1 for ACME indicate a significant mediation effect.

### Machine learning models

In the discovery cohort, candidate biomarkers were selected based on the differential analysis under the threshold FDR < 0.05 and FC > 1.2, with additional confounding adjustment for maternal age and gestational age. Significantly altered clinical parameters in EPL were also included for model development. The top-ranking features in the random forest (RF) model were determined by variable importance (%IncMSE) using 10-fold repeated cross-validation with *randomForestExplainer* (v0.10.1),^35^ while in logistic regression (LR) they were determined by a comprehensive evaluation for coefficient, *p*-value, and performance drop after feature removal with R package *stats* (v4.1.3) and *pROC* (v1.19.0.1).^36^^,^^37^ The multiomics and clinical data were randomly divided at a 7:3 ratio into a training dataset and a testing dataset. In the training dataset, RF algorithm was applied to build a multivariate model with R package *RandomForest* (v4.7-1.2).^38^ Moreover, to enhance the clinical interpretability, the components of the final RF model were used to fit an LR model with R package *pROC* (v1.19.0.1).^37^ The solo performance of key biomarkers was also investigated using LR models. Covariates (maternal age and gestational weeks) were adjusted in all models. In the testing dataset, the diagnostic performance was analysed by the receiver operating characteristics (ROC) analysis and AUC, sensitivity, specificity, positive predictive value (PPV), and negative predictive value (NPV). The optimal cut-off value of ROC was estimated using Youden's index. The difference of ROC curves between biomarkers or models was estimated by Delong test.^39^

In the validation cohort, features in the discriminative model were applied for EPL prediction by random survival forest (RSF) model with R package *randomForestSRC* (v3.5.1),^40^ with covariate adjustment. The data were randomly divided at a 7:3 ratio into a training dataset and a testing dataset. In the training dataset, patients were divided into high- and low-risk groups according to the ensemble risk score (ERS), and the cut-off value of ERS was defined as the median value of the training data. In addition, the EPL probability was estimated. Discretisation of features and hyperparameter tuning were performed. In the testing dataset, the concordance index (C-index) and AUC were evaluated for discrimination ability. Calibration curves were drawn to assess the goodness-of-fit between the nomogram-predicted probability and the actual proportion.^41^ An insignificant *p*-value in Hosmer–Lemeshow test and a lower Brier score indicate good model calibration. In addition, decision curve analysis was conducted to determine clinical utility by estimating the net benefits at different threshold probabilities with R package *dcurves* (v0.5.1).^42^

### Statistics

Statistical analyses were performed, and data were visualised by R software (version 4.1.3). The continuous variables and categorical variables were shown as mean ± standard deviation (SD) or count and its percentage (N, %), respectively. The demographic and clinical feature data of case and control groups were tested for normal distribution with the Kolmogorov–Smirnov test, and for homogeneity of variances with the Levene's test. The comparisons of the demographic and clinical feature data between EPL and HP groups were conducted and the p-values were calculated with a two-tailed Student's t-test with the R package *rstatix*.^28^ The potential impact of maternal age and gestational age on biomarker levels was adjusted for, and the adjusted *p*-values were calculated using the analysis of covariance (ANCOVA) method by fitting the general linear model with the R package *stats*.

The sample size was estimated based on the expected mean and standard deviation (SD) of serum concentration of PD-L1, a published biomarker associated with miscarriage in early pregnancy.^43^ To achieve the power (1-β) of 0.8 and 0.05 significance (α) with a ratio of 1:1, and at least 22 patients with EPL and 22 HP women were required, calculated using the Sample Size Calculator for Clinical Study by Cleveland Clinic (https://riskcalc.org/samplesize/).^44^

### Role of funders

The funding bodies were not involved in study design, data collection, data analysis and interpretation, manuscript preparation, or the decision to submit for publication.

## Results

### Characteristics of the participants

The clinical characteristics of patients with early pregnancy loss (EPL, *n* = 40) and healthy pregnancies (HP, *n* = 40) in the discovery cohort were shown in Table 1. We found that maternal age (*p =* 0.545), BMI (*p* = 0.318), and gestational week (*p* = 0.064) were comparable between the two groups. In comparison with healthy pregnancies, patients with EPL had 1.82-fold lower β-HCG levels (*p* = 0.012) and decreased platelet count (*p* = 0.034), in line with previous reports.^45^^,^^46^ Moreover, there was a significant reduction in white blood cell (WBC) count (*p* < 0.001) and neutrophil proportion (NEU%, *p* < 0.001), accompanied by a higher lymphocyte proportion (LYM%, *p* < 0.001) in the EPL group, indicating an impaired immunological response associated with EPL.

**Table 1:** Characteristics of the subjects in the discovery cohort.

	Healthy pregnancy	Early pregnancy loss	*p*-value
n	40	40	NA
Maternal age (Years)	30.750 ± 6.364	31.825 ± 9.192	0.5449
BMI	23.626 ± 0.920	22.571 ± 1.002	0.3175
Gestational week	9.925 ± 2.828	9.225 ± 1.414	0.0635
SBP	105.400 ± 1.414	109.625 ± 14.859	0.0503
DBP	69.675 ± 5.657	73.025 ± 9.192	<0.0001
Gravidity	2.300 ± 1.414	2.775 ± 2.828	0.1486
Parity	0.700 ± 0.707	0.825 ± 0.707	0.4142
Biochemical test			
WBC (×10^9^/L)	8.186 ± 0.445	5.792 ± 0.339	<0.0001
NEU (%)	72.741 ± 4.313	62.954 ± 1.202	<0.0001
LYM (%)	20.824 ± 7.071	30.377 ± 2.970	<0.0001
MON (%)	5.462 ± 2.475	5.400 ± 1.626	0.8881
RBC (×10^12^/L)	4.389 ± 0.148	4.453 ± 0.170	0.5537
HGB (g/L)	121.270 ± 17.678	122.77 ± 4.950	0.6196
MCH (pg)	27.786 ± 5.020	27.731 ± 0.071	0.9341
MCHC (g/L)	318.405 ± 24.042	321.457 ± 2.121	0.4141
PLT (×10^9^/L)	271.595 ± 62.933	236.771 ± 78.489	0.034
HCG (mIU/mL)	52402.425 ± 20967.130	28773.633 ± 3457.045	0.0124

The comparisons of the demographic and clinical feature data between EPL and HP groups were conducted and the *p*-values were calculated with a two-tailed Student's t-test.

Abbreviations: Systolic blood pressure (SBP), Diastolic blood pressure (DBP), White blood cell count (WBC), Neutrophil (NEU), Lymphocyte (LYM), Monocyte (MON), Red blood cell count (RBC), Haemoglobin (HGB), Mean corpuscular volume (MCV), Mean corpuscular haemoglobin (MCH), Mean corpuscular haemoglobin concentration (MCHC), Platelet count (PLT), Human chorionic gonadotropin (HCG).

### Altered immunoregulatory protein profile in EPL compared with healthy pregnancy

**Fig. 1:**
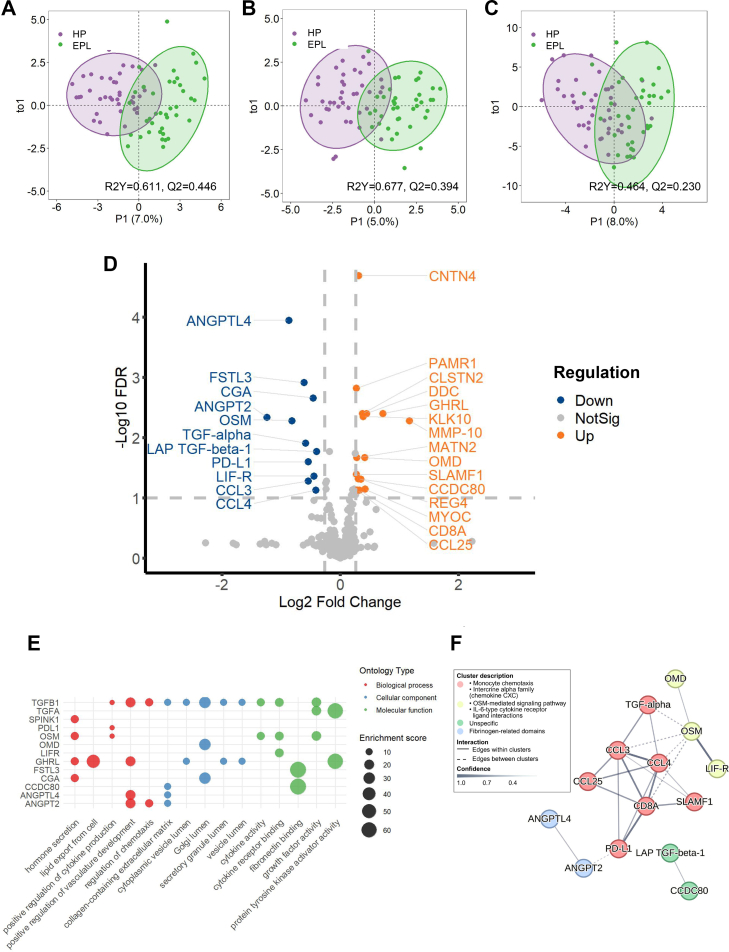
**Differential serum proteomic profiles in EPL and HP.** Serum protein profiling was conducted in 40 patients with EPL and 40 women with HP. OPLS-DA plot between early EPL and HP samples in (**A**) Olink Development Target 96 Panel, (**B**) Olink Inflammation Target 96 Panel, and (**C**) Olink Metabolism Target 96 Panel data. (**D**) Volcano plot of different DEP between EPL and HP groups, which were screened by threshold of FDR < 0.05 and FC > 1.2 after confounding factor adjustment, including maternal age and gestational weeks. (**E**) GO category analysis results of DEPs in EPL. (**F**) PPI of the DEPs in EPL. Clusters were denoted with different colours. Abbreviations: Osteomodulin (OMD), Matrilin-2 (MATN2), Transforming growth factor alpha (TGF-alpha, TGFA), Leukaemia inhibitory factor receptor (LIF-R), C-C motif chemokine 3 (CCL3), C-C motif chemokine 4 (CCL4), Inactive serine protease PAMR1 (PAMR1), Calsyntenin-2 (CLSTN2), Aromatic l-amino-acid decarboxylase (DDC), Appetite-regulating hormone (GHRL), Kallikrein-10 (KLK10), Coiled-coil domain-containing protein 80 (CCDC80), T-cell surface glycoprotein CD8 alpha chain (CD8A), Osteomodulin (OMD), Signalling lymphocytic activation molecule (SLAMF1), Myocilin (MYOC), Serine protease inhibitor Kazal-type 1 (SPINK1), Interleukin-6 (IL-6).

To delineate the protein profile in EPL, we employed Olink Development, Inflammation, and Metabolism Panels for the screening. There was significant diversity in circulating protein profiles between subjects with EPL and HP, as evidenced by the principal components analysis (PCA) (Supplementary Figure S2A–C). The orthogonal partial least squares-discriminant analysis (OPLS-DA) score plots further highlighted a marked separation between the two groups (Fig. 1A–C). After adjustment for confounders, including maternal age and gestational weeks, we screened 26 differently expressed proteins (DEPs) between the two groups from all three panels (fold change (FC) > 1.2, false discovery rate (FDR) < 0.05) (Fig. 1D, Table 2). Interestingly, angiopoietin-like 4 (ANGPTL4), a key protein with anti-inflammation properties and regulating lipid metabolism,^47^^,^^48^ was 1.85-fold decreased in EPL. Furthermore, several immune regulatory proteins were uniformly reduced in the EPL context, such as oncostatin M (OSM), programmed death-ligand 1 (PD-L1), angiopoietin 2 (ANGPT2), and follistatin-like 3 (FSLT3).^11^, ^49^, ^50^, ^51^, ^52^, ^53^ By contrast, a series of pro-inflammation proteins, such as matrix metalloproteinase 10 (MMP10), contactin-4 (CNTN4), and C-C motif chemokine (CCL25), were upregulated in EPL.^54^, ^55^, ^56^

**Table 2:** Significantly differently expressed proteins between EPL and HP.

Assay	*p*-value	FDR	FC	Adjusted *p*-value
Inflammation panel				
MMP-10	0.000111	0.005198	2.244982079	0.006379476
OSM	0.000113	0.005198	0.56673963	0.003591135
TGF-alpha	0.000398	0.012205333	0.665287788	0.003801179
PD-L1	0.0019	0.024971429	0.684466174	0.001597386
CCL3	0.00567	0.052164	0.685891758	0.010768478
LIF-R	0.00427	0.043648889	0.732725429	0.032149101
CCL4	0.0121	0.074213333	0.747744232	0.024251734
LAP TGF-beta-1	0.000918	0.0168912	0.755101303	0.003980774
CD8A	0.011	0.074213333	1.24783526	0.015996456
CCL25	0.0117	0.074213333	1.217711953	0.027754938
SLAMF1	0.00351	0.040365	1.204018605	0.029743299
Metabolism panel				
ANGPT2	0.00025	0.0046	0.422121615	0.005296311
GHRL	0.00013	0.003986667	1.644432223	0.002859297
DDC	9.04E-05	0.003986667	1.360833587	0.001522403
KLK10	0.000194	0.004462	1.308563996	0.002710172
CLSTN2	8.00E-05	0.003986667	1.295936199	0.002029415
REG4	0.00371	0.04876	1.273090099	0.021070095
CCDC80	0.00313	0.047993333	1.2325959	0.007397174
Development panel				
ANGPTL4	2.45E-06	0.0001127	0.546859926	0.000132022
FSTL3	3.97E-05	0.001217467	0.651560375	0.002638023
CGA	0.000119	0.0021896	0.727221209	0.003550906
MYOC	0.00629	0.071425455	1.337943937	0.015623939
OMD	0.00142	0.021422857	1.331948513	0.005358042
CNTN4	2.23E-07	2.05E-05	1.240395151	3.67917E-05
MATN2	0.00163	0.021422857	1.211817082	0.008830613
PAMR1	6.59E-05	0.0015157	1.203998327	0.000441988

The comparisons between EPL and HP groups were conducted and the *p*-values were calculated with a two-tailed Student's t-test. Maternal age and gestational weeks were adjusted using the analysis of covariance (ANCOVA) method. The Benjamini-Hochberg FDR method was applied to control for multiple testing.

Abbreviations:False discovery rate (FDR), Fold change (FC), Adjusted *p*-value (adj.*p*), Angiopoietin-related protein 4 (ANGPTL4), Follistatin-related protein 3 (FSTL3), Contactin-4 (CNTN4), Osteomodulin (OMD), Matrilin-2 (MATN2), Latency-associated peptide transforming growth factor beta-1 (LAP TGF-beta-1, TGFB1), Oncostatin M (OSM), Transforming growth factor alpha (TGF-alpha, TGFA), Leukaemia inhibitory factor receptor (LIF-R), Human plasma programmed cell death 1 ligand 1 (PD-L1), C-C motif chemokine 3 (CCL3), C-C motif chemokine 4 (CCL4), C-C motif chemokine 25 (CCL25), Inactive serine protease PAMR1 (PAMR1), Matrix metalloproteinase-10 (MMP-10), Angiopoietin-2 (ANGPT2), Calsyntenin-2 (CLSTN2), Aromatic l-amino-acid decarboxylase (DDC), Appetite-regulating hormone (GHRL), Kallikrein-10 (KLK10), Coiled-coil domain-containing protein 80 (CCDC80), T-cell surface glycoprotein CD8 alpha chain (CD8A), Osteomodulin (OMD), Signalling lymphocytic activation molecule (SLAMF1), Myocilin (MYOC), Regenerating islet-derived protein 4 (REG4), Serine protease inhibitor Kazal-type 1 (SPINK1), Glycoprotein hormones alpha chain (CGA).

We subsequently interrogated their functional difference based on screened DEPs. Gene Ontology (GO) analysis demonstrated an enrichment in positive regulation of cytokine production, cytokine activity, regulation of chemotaxis, and cytokine receptor binding, reflecting an aggravated immune status in EPL (Fig. 1E). Further protein–protein interaction (PPI) analysis revealed four centralised clusters and the majority of key proteins involved in monocyte chemotaxis (e.g., CCL family cytokines) and OSM signalling pathway, highlighting the importance of inflammation-mediated signalling in EPL pathology (Fig. 1F). Altogether, our proteomics identified several specific biomarkers and further unveiled an inflammatory and impaired tolerogenic status in EPL.

### Metabolomics reveals that EPL is featured by lipid metabolism dysfunction

**Fig. 2:**
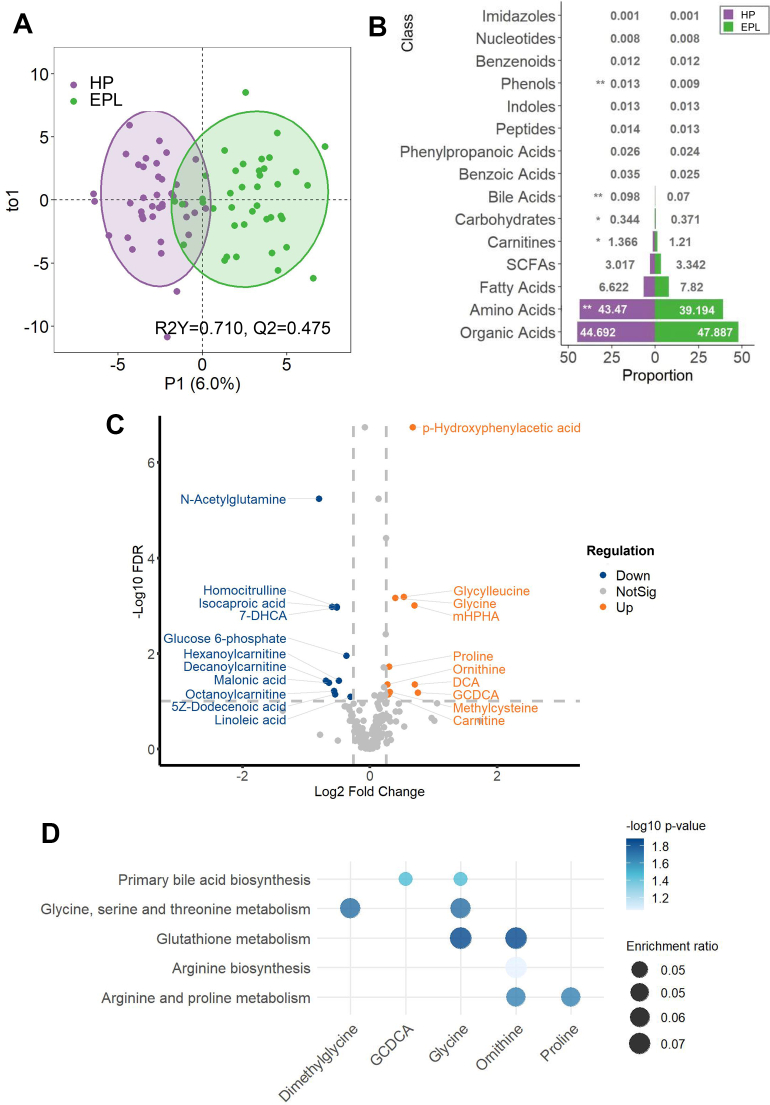
**Differential serum metabolic profiles in EPL and HP.** Serum metabolomics profiles were quantified using UPLC-MS/MS system in 40 patients with EPL and 40 women with HP. (**A**) OPLS-DA plot between EPL and HP samples. (**B**) Bar chart representing the proportion of circulating metabolites by class levels in EPL versus HP groups. Two-tailed Student's t-test was used for statistical comparisons. ∗*p*-value <0.05, ∗∗*p*-value <0.01. (**C**) Volcano plot of DEM between EPL and HP groups, which were screened by threshold of FDR < 0.05 and FC > 1.2 after confounding factor adjustment, including maternal age and gestational weeks. (**D**) Top five pathways in KEGG enrichment analysis associated with EPL. Abbreviations: 3-(3-Hydroxyphenyl)-3-hydroxypropanoic acid (mPHPA), Deoxycholic acid (DCA), glycochenodeoxycholate (GCDCA).

In terms of circulating metabolomics profiles in EPL, we quantified a total of 194 metabolites by UPLC-MS/MS methods for downstream analysis (Supplementary Table S2). Similarly, serum metabolite profiles were substantially separated between EPL and HP, as shown by the PCA plot (P < 0.05, Supplementary Figure S2D) and the OPLS-DA plot (R^2^Y = 0.710, Q^2^ = 0.475, Fig. 2A). Compared to the control group, the EPL group displayed significant decreases in phenols, bile acids, carnitines, and amino acids, whereas carbohydrates and organic acids were significantly elevated (Fig. 2B). We identified 21 metabolites after confounding adjustments with FDR < 0.05, FC > 1.2 (Fig. 2C, Table 3). Of note, N-Acetylglutamine, a glutamine derivative with immunoregulatory capacities and involved in glutathione synthesis, was the most downregulated metabolite in EPL.^57^ This result was further supported by the augmented glutathione metabolism pathway in KEGG enrichment analysis (Fig. 2D). Moreover, carnitine, an essential molecule for long-chain fatty acids (LCFA) transport and oxidation,^58^ was remarkably upregulated in the EPL group. By contrast, a series of metabolic intermediates of FA-conjugated carnitine, including octanoylcarnitine, decanoylcarnitine, and hexanoylcarnitine, were uniformly decreased. Given their well-established role in mitochondrial FA transport and oxidation,^59^ the simultaneous elevation of carnitine alongside reduced carnitine derivatives in EPL indicated a mitochondrial dysfunction as a potential underlying mechanism involved in EPL. Similarly, disturbances in polyunsaturated fatty acid (PUFA) metabolism were observed in EPL as evidenced by the reduction in levels of linoleic acid (LA), dodecenoic acid, and malonic acid (Fig. 2C). Altogether, the divergent metabolite profile implies an abnormal lipid oxidation and defective mitochondrial lipid metabolism associated with early pregnancy loss.

**Table 3:** Significantly altered metabolites in EPL compared with HP.

Metabolite	*p*-value	FDR	FC	Adjusted *p*-value
p-Hydroxyphenylacetic acid	1.87E-09	1.81E-07	1.594927878	3.97712E-05
N-Acetylglutamine	1.17E-07	5.67E-06	0.573463917	7.44388E-05
Glycylleucine	2.01E-05	0.0006499	1.446732735	0.001166263
Glycine	2.47E-05	0.000684543	1.31734122	0.001573666
3-(3-Hydroxyphenyl)-3-hydroxypropanoic acid	4.03E-05	0.000977275	1.621837585	0.00033736
Homocitrulline	4.92E-05	0.0010476	0.697871768	0.000365706
Isocaproic acid	5.40E-05	0.0010476	0.661807878	0.000211942
7-DHCA	6.16E-05	0.0010864	0.697898602	0.000722574
Glucose 6-phosphate	0.00074	0.011043077	0.772160675	0.002980645
Proline	0.00137	0.018984286	1.231007474	0.005645391
Decanoylcarnitine	0.00322	0.036974118	0.618605222	0.007041163
Hexanoylcarnitine	0.00324	0.036974118	0.711364606	0.005983929
Malonic acid	0.0038	0.040955556	0.639128042	0.011030188
DCA	0.0046	0.044717	1.628098489	0.010759056
Ornithine	0.00461	0.044717	1.208341252	0.022090661
Octanoylcarnitine	0.00693	0.06111	0.676934825	0.012993691
Methylcysteine	0.00755	0.063682609	1.2431383	0.022106649
GCDCA	0.00819	0.0662025	1.686165551	0.027607041
Carnitine	0.0093	0.071705385	1.223453228	0.017848156
5Z-Dodecenoic acid	0.00961	0.071705385	0.683897853	0.013523529
Linoleic acid	0.0125	0.080833333	0.808017553	0.007532139

The comparisons between early pregnancy loss and healthy pregnancy groups were conducted and the *p*-values were calculated with a two-tailed Student's t-test. Maternal age and gestational weeks were adjusted using the analysis of covariance (ANCOVA) method. The Benjamini-Hochberg false discovery rate (FDR) method was applied to control for multiple testing.

Abbreviations: False discovery rate (FDR), Fold change (FC), Adjusted *p*-value (adj.*p*), 3-(3-Hydroxyphenyl)-3-hydroxypropanoic acid (mPHPA), Deoxycholic acid (DCA), glycochenodeoxycholate (GCDCA), 7-Deoxycholic acid (7-DHCA).

### Circulating protein-metabolites interaction patterns are distinct between EPL and HP

**Fig. 3:**
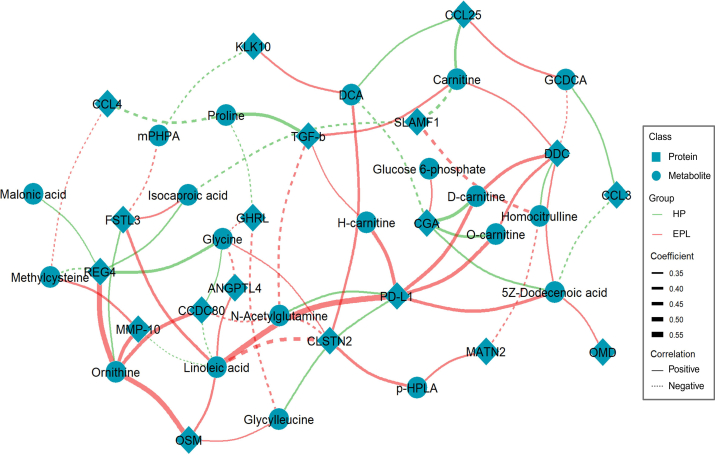
**Interaction network of the circulating proteins and metabolites in EPL and HP.** In 40 patients with EPL and 40 women with HP, partial correlation of the 26 DEPs and 21 DEMs was conducted for their association in EPL and HP, respectively. Covariates (maternal age and gestational weeks) were adjusted in the analysis. Among the identical node positions, the red edges denote interactions in EPL group, while the green edges denote the interactions in HP group. Only significant correlations (*p*-value <0.05) in partial correlation analysis were demonstrated in the interaction network. Abbreviations: p-Hydroxyphenylacetic acid (p-HPLA), Hexanoylcarnitine (H-carnitine), Octanoylcarnitine (O-carnitine), Decanoylcarnitine (d-carnitine).

Given the distinct omics patterns in EPL and healthy pregnancy, we next performed a partial correlation analysis and found a striking difference in protein-metabolite interaction network between the two groups. As shown in Fig. 3 and Supplementary Figure S3, in subjects with EPL, reduced PD-L1, LA, and ornithine emerged as central protein hubs extensively interconnected with metabolites. Given their immune-suppressive effects,^60^, ^61^, ^62^ these interactions indicated an aggravated immune status and corresponding metabolic adaptation in EPL pathology. In healthy pregnancy, the metabolite-protein network exhibited a concentrated network centralised by glycoprotein hormones alpha chain (CGA), proline, and regenerating islet-derived protein 4 (REG4), which are essential modulators in mitochondrial oxidation and agents with anti-apoptotic properties.^63^, ^64^, ^65^ Altogether, our integrative analysis suggested synergetic lipid–immune interactions for pregnancy loss, and underscored the importance of mitochondrial function and proliferation for pregnancy maintenance.

### Serum biomarker levels are associated with clinical phenotypes in EPL

**Fig. 4:**
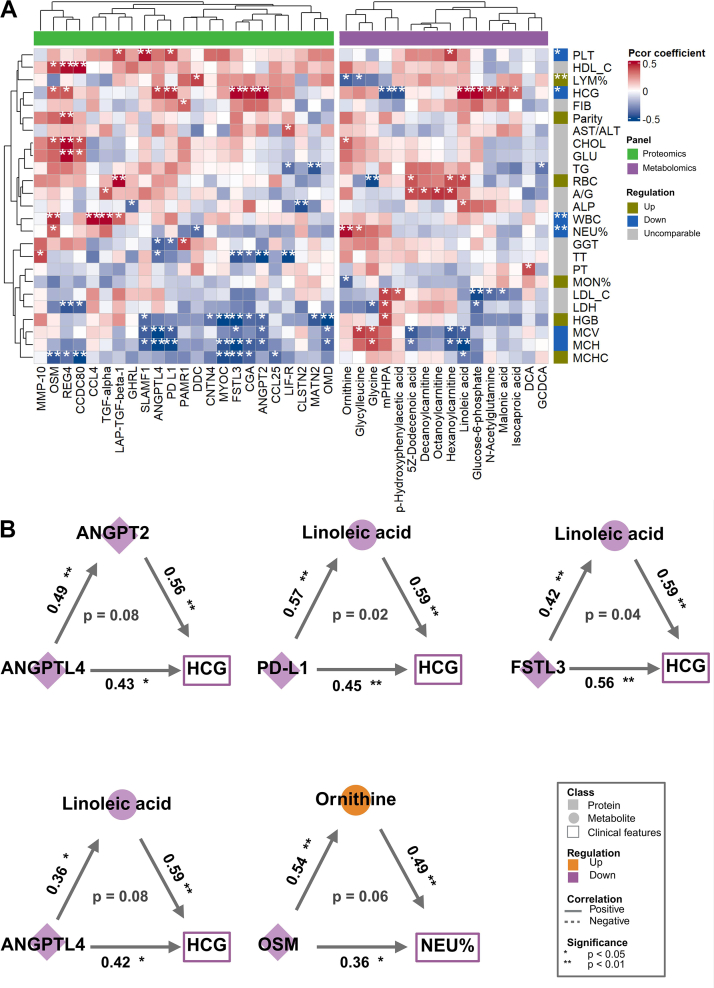
**Correlation analysis between screened proteins and metabolites and clinical features in patients with EPL.** Analyses of the 40 EPL patients included **(A)** a heatmap depicting partial correlations between screened biomarkers and clinical parameters, and **(B)** mediation analysis examining proteins, metabolites, and clinical features. The figures on the edge demonstrate the coefficient of partial correlation analysis between the vertices, and figures in the middle of the triangle demonstrate the significance of the mediation effect. Covariates (maternal age and gestational weeks) were adjusted in the analysis using ANCOVA method. ∗*p*-value <0.05, ∗∗*p*-value <0.01. Abbreviations: Systolic blood pressure (SBP), Diastolic blood pressure (DBP), Albumin/Globulin ratio (A/G), Alanine aminotransferase (ALT), Aspartate aminotransferase (AST), Lactate dehydrogenase (LDH), Cholesterol (CHOL), Triglycerides (TG), High-density lipoprotein cholesterol (HDL-C), Low density lipoprotein cholesterol (LDL-C), Glucose (GLU), Monocyte (MON), Red blood cell count (RBC), Haemoglobin (HGB), Mean corpuscular volume (MCV), Mean corpuscular haemoglobin (MCH), Mean corpuscular haemoglobin concentration (MCHC), Platelet count (PLT), Thrombin time (TT), Prothrombin time (PT), Fibrinogen (FIB), Partial correlation (pcor).

To illustrate the possible contribution of altered proteins and metabolites to EPL, we conducted a partial correlation analysis to evaluate their association with clinical parameters lasts in early pregnancy to stimulate trophoblast invasion and differentiation,^66^ where lower β-HCG levels are widely observed in EPL status. In our study, we found ANGPTL4, OSM, PD-L1, ANGPT2, FSTL3, and CGA, together with key metabolites, such as N-Acetylglutamine and LA, were positively correlated with the β-HCG level in patients with EPL (Fig. 4A). Since β-HCG is mainly secreted from the trophoblast, this coordinated molecular linkage suggested a possible synchronised metabolic-immune regulatory axis in mediating β-HCG production and trophoblast cell function during early pregnancy failure. Moreover, aligned with the infection and inflammation stress that occurred in EPL,^67^ we found that the decreased OSM level was correlated with decreased WBC count and NEU% in our study. In addition, ANGPTL4 and ANGPT2 were negatively correlated with thrombin time, which is consistent with their fibrinogen-related domains in PPI results (Fig. 1F). Meanwhile, PD-L1 was positively associated with platelet count in EPL. Interestingly, abnormal platelet counts were observed in women with EPL.^68^ Given the effects of PD-L1 on platelet activation,^69^ our data implied that coagulation dysfunction may contribute to EPL. Overall, our candidates are tightly associated with the known aetiology for EPL, especially in terms of β-HCG concentration, immune status, and coagulation conditions.

Given the extensive correlations between EPL-specific biomarkers and clinical indexes, we further applied mediation analysis to clarify their potential interplay (Fig. 4B). In line with the biomarkers-β-HCG linkages unravelled in correlation analysis (Supplementary Figure S4), we observed several novel regulatory signalling may determine β-HCG production. The presence of ANGPTL4 (*r* = 0.43, *p* = 0.02) and ANGPT2 (*r* = 0.56, *p* < 0.001) positively contributed to serum β-HCG. Moreover, PD-L1 (*r* = 0.45, *p* = 0.01) and FSTL3 (*r* = 0.56, *p* < 0.001) also showed synergic effects on β-HCG. Importantly, LA is not only positively correlated with β-HCG level (*r* = 0.59, *p* < 0.001), but also a critical molecular transducer mediating the effect of ANGPTL4, PD-L1, and FSTL3 on β-HCG production (*p* = 0.08, *p* = 0.02, *p* = 0.04, respectively). Moreover, a decreased NEU% in EPL cases was positively linked with OSM (*r* = 0.36, *p* = 0.045), supported by previous reports that OSM is a selective chemoattractant of neutrophils during inflammatory response.^70^ Interestingly, this effect was remarkably mediated by ornithine (*p* = 0.06, IE = 0.12 (95% CI = (0.00–0.33)), a metabolite with immune regulatory effects on leucocytes.^71^ Taken together, our data identified several central regulators that may be associated with β-HCG production and serum neutrophil% in EPL.

### Biomarkers screened by multi-omics strategies effectively distinguished EPL from HP

**Fig. 5:**
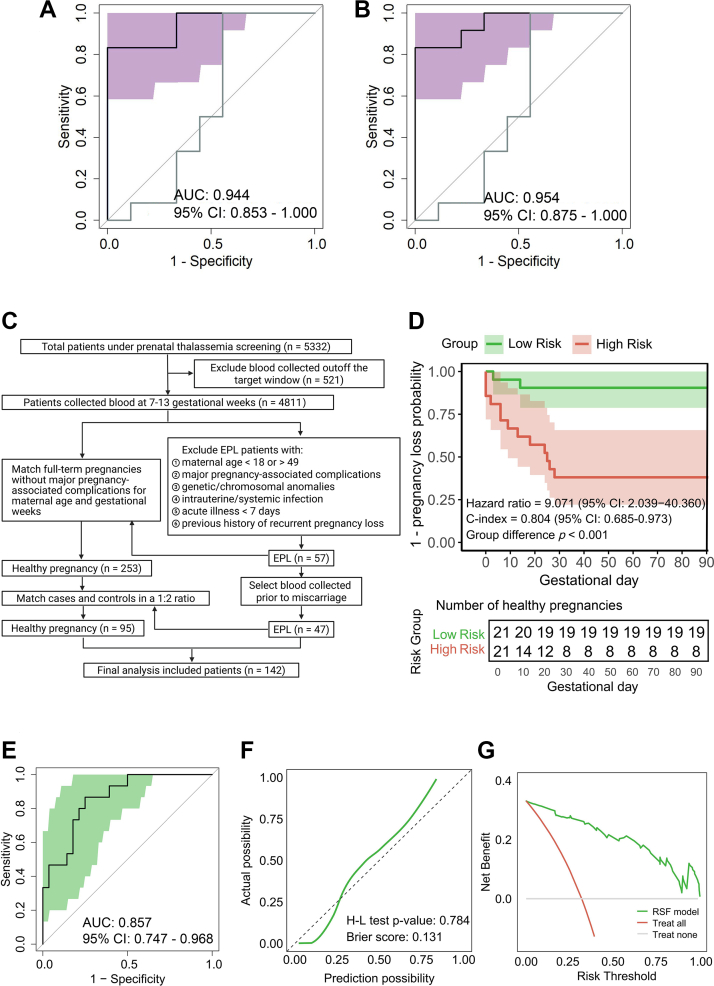
**Development and prospective validation of the 4-variable models (ANGPTL4, PD-L1, NEU%, LYM%) for EPL prediction.** Multi-omics and clinical data from 40 patients with EPL and 40 women with HP in the discovery cohort were applied to develop the discriminative model, which was further validated in the prospective cohort, including 47 patients with EPL and 95 women with HP. ROC plots of (**A**) RF model and (**B**) LR model in the discovery cohort to distinguish EPL from HP. The ROC of β-HCG was shown as the grey curve. Shaded areas represent 95% CI of the AUC estimates. (**C**) Schematic enrolment flowchart for the external validation cohort. (**D**) Survival analysis of the RSF model in the validation cohort. (**E**) ROC plot of the RSF model in the validation cohort to predict EPL. (**F)** Calibration curve of the RSF model. (**G**) Decision curve analysis of the RSF model. The features in RF, LR, and RSF models are ANGPTL4, PD-L1, NEU%, and LYM%.

Lastly, we developed the discriminative model for EPL based on screened biomarker candidates. We employed a random forest (RF) approach to rank the importance of all candidate variables, which yielded an initial list of the ten most important biomarkers (Supplementary Figure S5A). Subsequent stepwise selection procedure narrowed the model down to four pivotal biomarkers, including ANGPTL4, PD-L1, NEU%, and LYM% (Supplementary Table S3). Interestingly, this four-biomarker set was identical to the top four features established in logistic regression (LR) analysis (Supplementary Figure S5B, Supplementary Table S4), indicating a model-agnostic stability and general biological and clinical relevance of the biomarker panel for EPL. We then built discriminative models based on these four biomarkers using both the RF and LR methods. After controlling maternal age and gestational week, both models demonstrated excellent ability to differentiate EPL cases from normal pregnancies (Fig. 5A), with the RF model attaining an AUC of 0.944 (95% CI: 0.853–1) and the LR model attaining an AUC of 0.954 (95% CI: 0.875–1) (Fig. 5B). By contrast, β-HCG is the most widely applied clinical biochemistry index for EPL identification. However, it only achieved an AUC of 0.556 (95% CI: 0.253–0.858). Importantly, the four-candidate-based model outperformed β-HCG (*p* = 0.024, *p* = 0.007) and any single biomarker (Table 4, Supplementary Table S5), underscoring their robustness and the translational potential in EPL diagnostics.

**Table 4:** Performance of the biomarkers and models in differentiating EPL from HP.

Biomarker	AUC	95% CI	Sensitivity	Specificity	Threshold	PPV	NPV	*p*-value
HCG	0.556	0.253–0.858	1.000	0.444	0.287	0.705	1.000	NA
PD-L1	0.796	0.572–1.000	0.833	0.778	0.451	0.833	0.778	0.081
ANGPTL4	0.833	0.655–1.000	0.583	1.000	0.745	1.000	0.642	0.068
RF model	0.944	0.835–1.000	0.833	1.000	0.548	1.000	0.818	0.024
LR model	0.954	0.853–1.000	0.833	1.000	0.552	1.000	0.818	0.007
RSF model	0.857	0.747–0.968	0.800	0.786	3.986	0.650	0.913	NA

RF, LR, and RSF models consist ANGPTL4, NEU%, LYM%, and PD-L1. Maternal age and gestational weeks were adjusted using the analysis of covariance (ANCOVA) method. The difference of ROC curves between biomarkers or models was estimated by Delong test. *P*-value implies the significance of the detective difference between the biomarker or model and HCG.

Abbreviations: Area under receiver operating characteristic curve (AUC), Positive predictive value (PPV), Negative predictive value (NPV),Angiopoietin-related protein 4 (ANGPTL4), Human plasma programmed cell death 1 ligand 1 (PD-L1), Human chorionic gonadotropin (HCG), Neutrophil (NEU), Lymphocyte (LYM), Random forest (RF), Logistic regression (LR), Random survival forest (RSF).

### Applying serum levels of NEU%, LYM%, ANGPTL4, PD-L1 predicts the onset of EPL

To further ascertain our results, we recruited an independent cohort of 142 pregnant women at 7–10 weeks of gestation. Among them, 47 subjects experienced pregnancy loss at 8–12^+6/7^ weeks of gestation, and 95 HP with full-term births without any major gestational diseases (Fig. 5C). The maternal age and gestational week were comparable between the two groups (Table 5). In line with the discovery cohort, NEU% was 4.8% increased, whereas LYM% was 11.2% decreased in the EPL group. Importantly, serum ANGPTL4 and sPD-L1 levels were around 2-fold lower in the EPL group, and the differences remain robust after adjusting for confounders (Table 5).

**Table 5:** Characteristics of the subjects in the validation cohort.

	Early pregnancy loss	Healthy pregnancy	*p*-value
n	47	95	NA
Gestational day	60.400 ± 8.960	61.830 ± 9.913	0.408
Maternal age (Years)	31.100 ± 4.287	30.160 ± 3.248	0.146
Terminal of pregnancy after sample collection (day)	13.638 ± 10.180	211.170 ± 9.913	<0.001
Biochemical test			
WBC (×10^9^/L)	8.430 ± 1.942	8.557 ± 1.643	0.684
NEU (%)	68.280 ± 5.345	71.580 ± 4.772	<0.001
LYM (%)	24.230 ± 4.549	21.510 ± 4.193	<0.001
RBC (×10^12^/L)	4.350 ± 0.356	4.272 ± 0.436	0.288
HGB (g/L)	129.500 ± 8.851	124.600 ± 10.600	0.007
PLT (×10^9^/L)	260.800 ± 64.280	250.500 ± 58.530	0.343
Serum target biomarker concentration (adjusted for maternal age and gestational days)			
PD-L1 (pg/mL)	28.869 ± 7.837	58.208 ± 17.642	<0.001
Adjusted PD-L1 (pg/mL)	28.553 ± 9.010	58.973 ± 18.752	<0.001
ANGPTL4 (ng/mL)	4.695 ± 3.486	9.165 ± 7.954	0.003
Adjusted ANGPTL4 (ng/mL)	5.099 ± 4.843	10.421 ± 11.063	0.002

The comparisons of the demographic and clinical feature data between EPL and HP groups were conducted and the *p*-values were calculated with a two-tailed Student's t-test.

Abbreviations: White blood cell count (WBC), Neutrophil (NEU), Lymphocyte (LYM), Red blood cell count (RBC), Haemoglobin (HGB), Platelet count (PLT), Programmed death-ligand 1 (PD-L1), angiopoietin-like 4 (ANGPTL4).

Next, we used the four markers (NEU%, LYM%, ANGPTL4, PD-L1) to construct a predictive model using a random survival forest (RSF) approach, which achieved an AUC of 0.857 (95% CI: 0.747–0.968) and concordance index (C-index) of 0.804 (95% CI: 0.685–0.973) after confounder adjustment (Fig. 5D and E). We further evaluated the model's clinical utility by defining a risk threshold (ensemble risk score, ERS = 5.515) to categorise women into high-risk and low-risk groups. Strikingly, about 61.9% of women in the high-risk category experienced an EPL within 30 days after sampling, in contrast to only 9.5% in the low-risk category (Fig. 5D, *p* < 0.001). Of note, our prediction model was also well-calibrated, as shown by predicted risks closely matched observed outcomes (Hosmer–Lemeshow test *p* = 0.784; Brier score 0.131, Fig. 5F). Moreover, decision curve analysis indicated that the model offers a net benefit in guiding interventions across a broad range of risk thresholds. Applying the model could help clinicians identify approximately 20 additional high-risk cases per 100 pregnancies for preventative measures at a 50% risk threshold, compared to the default strategy of not using the model (Fig. 5G). In summary, this prospective validation demonstrates that our four-biomarker panel retains robust predictive value for EPL, enabling early identification of high-risk pregnancies.

## Discussion

Our multi-omics screening identified 26 proteins and 21 metabolite biomarkers significantly altered in the blood samples of EPL. Moreover, the integrative analysis indicated several potential mechanistic signalling implicated in EPL, potentially acting through the dysregulation of β-HCG production, immune response, and lipid metabolism. Of note, applying serum levels of ANGPTL4, PD-L1, and clinical parameters potentially provides a non-invasive approach for early identification and risk stratification for EPL.

Successful pregnancy requires finely adapted immune homoeostasis and immunologic privilege status in mothers,^72^ whereas inflammation and hyperimmune activation contribute to pregnancy loss.^73^ Our results revealed that a cluster of immunosuppressive proteins was uniformly reduced in EPL, including ANGPTL4, PD-L1, OSM (a pleiotropic cytokine belonging to IL-6 family cytokine family^49^), ANGPT2 (a protein reducing immune response and increasing PD-L1 expression on M2-polarised macrophages^52^), and FSLT3 (a primarily antagonist inhibiting transforming growth factor-β (TGF-β) family cytokines^53^). Conversely, pro-inflammation proteins, like MMP10 (an activator of macrophage migration and invasion^54^), CNTN4 (a mediator of lipid-induced mast cell hyper-reactivity^55^), and CCL25 (a member of the CC subfamily of chemokines promoting a variety of inflammatory diseases^56^) were upregulated in EPL. Parallelly, decreases in immunosuppressive metabolites were observed in metabolomics data. Malonic acid is 1.5-fold lower in EPL cases, which was reported as immunosuppressants that inhibit NF-κB activation.^74^ By contrast, metabolites with immune-stimulating functions, such as carnitine (reduces lymphocyte apoptosis and improve inadequate inflammatory response in infection^75^), ornithine (a polyamines precursor with T-cell regulation functions,^61^ promoting cytokine storm in infection^76^), and proline (increases leucocyte population and cytokine production^77^), were consistently increased in EPL. These findings implicated pathogenesis of EPL is highly relevant to the inflammatory process and immune dysfunction.

By combining the metabolomics approach, our study delineates an EPL-associated metabolic signature and its possible underlying mechanism. Carnitine-related metabolites formed one of the central metabolic clusters and were linked to CGA, the β-hCG subunit,^78^ which is consistent with the regulatory role of carnitines in mitochondrial activity and bioenergetic homoeostasis for healthy embryo development.^63^ Moreover, we found that proline is positively correlated with TGF-beta in normal pregnancy. Given that TGF-β mediates the proline biosynthesis from glutamine in mitochondrial oxidation,^65^ alterations in proline levels between EPL and HP may reflect dysregulated TGF-β–proline axis activity, with implications for trophoblast survival and tissue homoeostasis. Alternatively, increased ornithine was positively correlated with pro-inflammatory proteins such as MMP10 and coiled-coil domain-containing protein 80 (CCDC80), indicating a metabolic adaptation to eliminate the abnormal immune activation. Interestingly, we observed that serum LA levels are significantly reduced in EPL cases. In the context of gestation, LA and its derivative forms, conjugated linoleic acid (CLA), play important roles in facilitating implantation and coordinating trophoblast functions. For instance, LA stimulation inhibits pro-inflammatory cytokines IL-6 but increases anti-inflammatory IL-8 expression in human trophoblast cells.^79^ Likewise, CLA treatment induced the pro-angiogenic gene expression and tube formation in HTR8/SVneo cells,^80^ suggesting its beneficial effects in the early placentation process. Moreover, CLA also significantly induces ANGTPL4 expression in trophoblasts,^80^ which agrees with our mediation analysis, where an assumed interplay between ANGTPL4 and LA. Together, our findings emphasise that metabolite disturbances, alongside immunologic alterations, provide crucial mechanistic clues to EPL pathophysiology.

ANGPTL4 is a multifunctional protein involved in the regulation of lipid metabolism, glucose homoeostasis, inflammation, angiogenesis, and vascular permeability.^81^ In the context of gestation, decreased serum levels of ANGPTL4 have been observed in patients with recurrent implantation failure,^82^ selective intrauterine growth restriction,^83^ and preeclampsia.^84^ Our data demonstrated that serum ANGPTL4 levels were around 50% lower in EPL cases in both the discovery and validation cohorts, which is further in line with previous reports and implies the important role of ANGPTL4 in pregnancy health. Mechanistically, ANGPTL4 enhances placental explant outgrowth and angiogenesis in human umbilical vein endothelial cells.^84^ In vitro studies showed ANGPTL4 mediates trophoblast cell survival, proliferation, migration, and invasion through G protein-coupled oestrogen receptor (GPER) and PPAR*γ*-dependent signalling.^84^^,^^85^ Our integrative omics analysis further unearthed a significant correlation between β-HCG and ANGPTL4, indicating a ANGPTL4 may also contribute to pregnancy failure by regulating β-HCG.

PD-L1 comprises an important inhibitory checkpoint signalling system for immune regulation.^86^ During gestation, serum soluble PD-L1 level (sPD-L1) is increased compared to non-pregnant women,^87^ and progressively increases with gestational weeks.^43^ Our previous study found decreased PD-L1 levels in both serum and placenta samples in patients with miscarriage.^11^ Importantly, applying serum PD-L1 resulted in an AUC of 0.73 to predict miscarriage two weeks in advance in women with in vitro fertilisation (IVF).^11^ Consistently, our data from the natural pregnancy cohort also found a 50% decrease in patients who subsequently developed EPL compared with healthy pregnancies with full-term birth at 7–10 weeks of gestation. These concordant findings from both assisted and spontaneous conceptions suggest circulating PD-L1 levels as a generalisable biomarker with a robust early detection capacity of pregnancy loss. Furthermore, decreased PD-L1 exhibited in EPL positively correlated with various carnitine derivatives, such as d-carnitine and O-carnitine, which is in line with reports that carnitine treatment significantly increased cell surface PD-L1 expression in acute monocytic leukaemia cell lines.^88^ Moreover, PD-L1 was positively linked with decreased LA levels in EPL. Overall, these correlations suggest a plausible immunometabolism axis whereby PD-L1 intersects with metabolites in EPL.

Our study presented several advantages. First, by combining advanced proteomics and metabolomics with clinical information, we established an integrative, multi-omics profiling for blood-based biomarker discovery in EPL. Compared to previous biomarker screening conducted in decidua, villi tissues, or endometrium tissue for miscarriage,^89^, ^90^, ^91^ our data from serum samples offer non-invasive biomarkers for EPL. Importantly, we employed widely adopted strategies for integrative multi-omics analysis, allowing us to reveal dynamic interaction between immune and metabolic markers and their influence on clinical phenotypes and providing a comprehensive molecular landscape into the pathophysiology of EPL. Another advantage in study design is that all healthy pregnancies in the control group were followed until delivery without any major complications. Moreover, we carefully matched the maternal age and gestational weeks between the two groups and further adjusted the potential confounding factors throughout the integrative analysis to ensure the analytical rigour and the reliability of multi-omics screening. Lastly, we developed EPL models using blood-based markers and clinical parameters, which were further validated in a prospective cohort with predictive value.

Meanwhile, several limitations of our study warrant consideration. First, although we applied a two-stage, discovery-to-validation strategy for omics screening, the sample size in our discovery phase was still relatively limited, which may reduce the reduced biomarkers detecting sensitivity and constrain fine-grained subgroup analyses to fully capture EPL. Second, our results were conducted in a Chinese population, multi-ethnic validation will be required to confirm generalisability across diverse population and clinical settings. Third, despite rigid rigorous participant selection and multivariable adjustment for major confounders, residual external factors may still influence blood biomarker profiles. In particular, subclinical or undetected infections during pregnancy could modulate inflammatory protein patterns. Moreover, inter-individual variability in metabolic status, such as differences in dietary intake or metabolic adaptation during early gestation, also influences circulating metabolomic profiles. Lastly, given the nature of the observational design, the cause-and-effect relationship between identified biomarkers and EPL required further experimental investigation. Further large-scall, multicenter, prospective cohorts with rigorous study designs are necessary to further estimate the model performance.

By integrating proteomics and metabolomics, our multiomics-based screening identified biomarker candidates for EPL at early pregnancy. Our integrative omics analysis uncovers multidimensional linkages between circulating effectors and miscarriage, offering plausible mechanistic insights into the pathology of pregnancy failure, including dysfunctions in immune regulation and oxidative-related lipid metabolism. Importantly, key molecules, such as ANGPTL4 and PD-L1, may regulate trophoblast activity and β-HCG production, suggesting possible targets for miscarriage management. Importantly, machine learning models incorporating circulating levels of ANGPTL4, PD-L1, NEU%, and LYM% are promising tools for early identification of EPL. Our data have translational feasibility to incorporate into clinical implementation for early identification and risk stratification, allowing for timely interventions and personalised care plans, and potentially improving the pregnancy outcomes.

## Contributors

Conceptualisation: Y.W., Methodology: Y.S., Y.W., and N.Y., Data curation: Y.Y., X.G., S.S., Q.L., and X.C., Formal analysis, Investigation, Visualisation, Software: Y.S., Y.W., and N.Y., Resources: Y.W., Y.Y., Q.L., and X.C., Validation: Y.S., Y.W., and X.L., Supervision: Y.W., Y.Y., and C.C.W., Writing-original draft: Y.S. and Y.W., Writing-review & editing: N.Y., Y.F.W., X.L., L.C.P., P.W.C., and J.X., Funding acquisition: Y.W. and X.L., Project administration: Y.Y., Q.L., and X.C. All authors read and approved the final version of the manuscript. All authors had full access to the data, and data verification was completed by Y.W., Y.Y., Q.L., and X.C.

## Data sharing statement

Reasonable requests for clinical or preclinical data not already shared in the manuscript or Supplementary File, can be directed to the corresponding author. Clinical data sharing will be conducted in accordance with Institutional Review Board approval. The custom code and scripts used for machine learning analyses are available from the corresponding author upon reasonable request.

## Declaration of interests

The authors declare that the research was conducted in the absence of any commercial or financial relationships that could be construed as a potential conflict of interest.

## Appendix A Supplementary data

Supplementary Figures and Tables

Reagent Validation
