# Supplementary material for: Blood-based biomarker discovery for early pregnancy loss using integrative multi-omics strategies
Source: eBioMedicine. 2026 Apr 13;127:106253. doi: 10.1016/j.ebiom.2026.106253 (PMC13092690; doi:10.1016/j.ebiom.2026.106253)
Supplement: Supplementary Figures and Tables [file mmc1.pdf]

Supplementary Data

Applying Multi-omics Approach and Integrative Analysis for Biomarker Discovery in Pregnancy Loss during Early Pregnancy

Yue SHI, Yongkang YANG, et.al.

Correspondence:
yueqiong.ni@connect.hku.hk (Y.N.);
chenxiaoyan@cuhk.edu.hk (X.C.);
yaowang1@cuhk.edu.hk (Y.W.)

This PDF file includes:

Supplementary figure 1: Page 2
Supplementary figure 2: Page 3
Supplementary figure 3: Page 4
Supplementary figure 4: Page 5-6
Supplementary figure 5: Page 7
Supplementary table 1: Page 8-12
Supplementary table 2: Page 13-14
Supplementary table 3: Page 15
Supplementary table 4: Page 16
Supplementary table 5: Page 17

Supplementary Figure 1

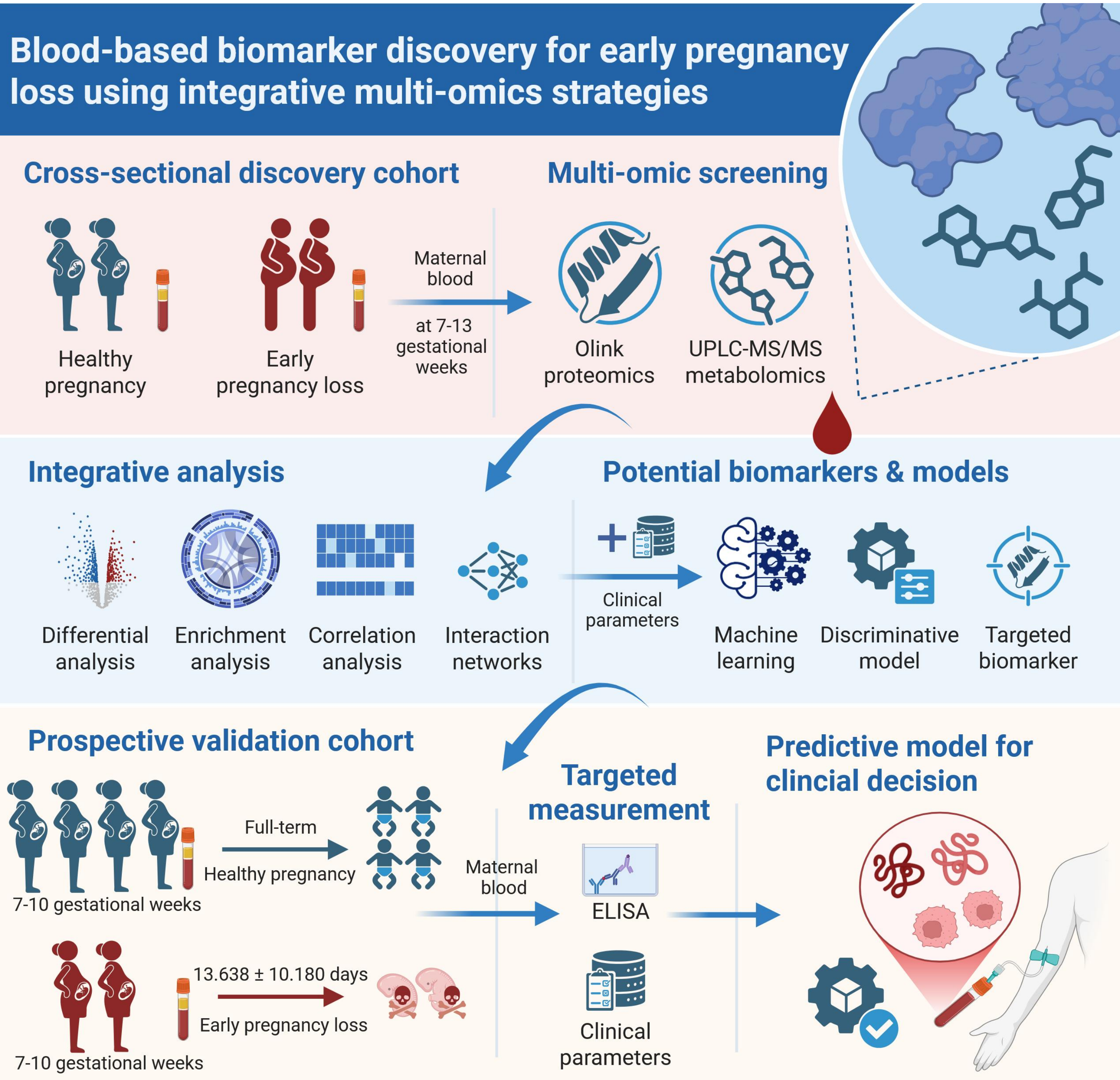

Supplementary Figure 1:Graphical abstract We organized a nested case-control study of women with early pregnancy loss (EPL) and maternal and gestational age-matched healthy pregnancies (HP) at 7-13 weeks of gestation for multi-omics screening to profile the metabolite and protein biomarkers. We further conducted integrative analysis to contextualize their putative contributions and interactions to EPL pathophysiology. The screened biomarkers were then validated in an independent perspective cohort with patients who subsequently developed EPL. Abbreviation: Enzyme-linked immunosorbent assay (ELISA), Ultra-performance liquid chromatography coupled to tandem mass spectrometry (UPLC-MS/MS)

Supplementary Figure 2

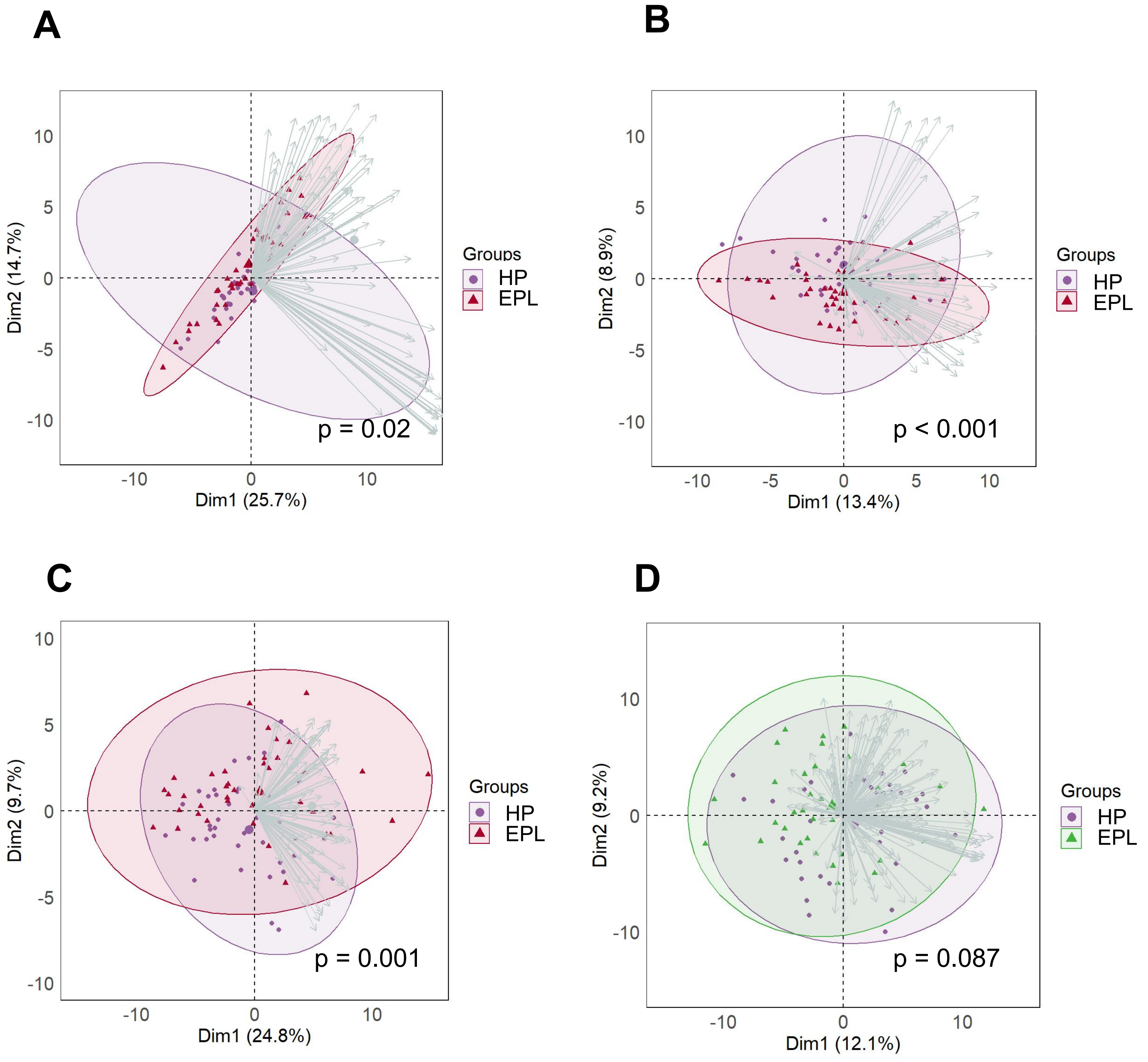

Supplementary Figure 2 : Principal component analysis (PCA) of the integrated omics data. Principal components analysis (PCA) plot of (**A**) Olink Development Target 96 Panel, (**B**) Olink Inflammation Target 96 Panel, (**C**) Olink Metabolism Target 96 Panel, and (**D**) ultra-performance liquid chromatography coupled to tandem mass spectrometry (UPLC-MS/MS) metabolite profile between early pregnancy loss (EPL) and healthy pregnancy (HP).

Supplementary Figure 3

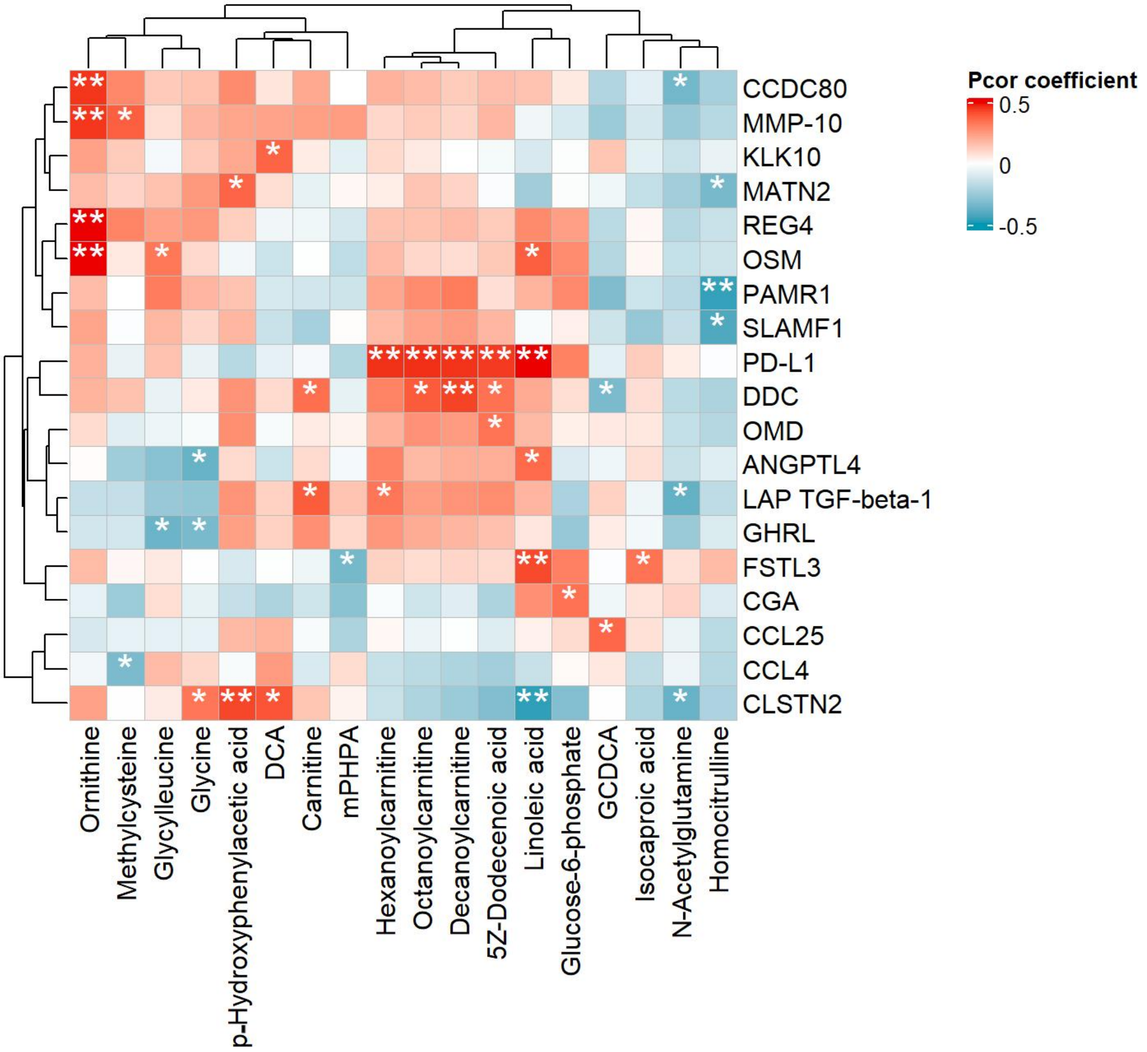

Supplementary Figure 3: Correlation between key proteins and key metabolites in patients with early pregnancy loss. Partial correlation of the 26 different expressed proteins (DEP) and 21 different expressed metabolites (DEM) was conducted for their association in patients with early pregnancy loss (EPL). Covariates (maternal age and gestational weeks) were adjusted in the analysis. *p-value < 0.05, ** p-value < 0.01. Abbreviation: Angiopoietin-related protein 4 (ANGPTL4), Follistatin-related protein 3 (FSTL3), Contactin-4 (CNTN4), Osteomodulin (OMD), Matrilin-2 (MATN2), Latency-associated peptide transforming growth factor beta-1 (LAP TGF-beta-1, TGFB1), Oncostatin M (OSM), Leukemia inhibitory factor receptor (LIF-R), Programmed cell death 1 ligand 1 (PD-L1), C-C motif chemokine 4 (CCL4), C-C motif chemokine 25 (CCL25), Inactive serine protease PAMR1 (PAMR1), Matrix metalloproteinase-10 (MMP-10), Calsyntenin-2 (CLSTN2), Aromatic L-amino-acid decarboxylase (DDC), Appetite-regulating hormone (GHRL), Kallikrein-10 (KLK10), Coiled-coil domain-containing protein 80 (CCDC80), T-cell surface glycoprotein CD8 alpha chain (CD8A), Osteomodulin (OMD), Signaling lymphocytic activation molecule (SLAMF1), Regenerating islet-derived protein 4 (REG4), Serine protease inhibitor Kazal-type 1 (SPINK1), 3-(3-Hydroxyphenyl)-3-hydroxypropanoic acid (mPHPA), Deoxycholic acid (DCA), glycochenodeoxycholate (GCDCA), Partial correlation (pcor).

Supplementary Figure 4

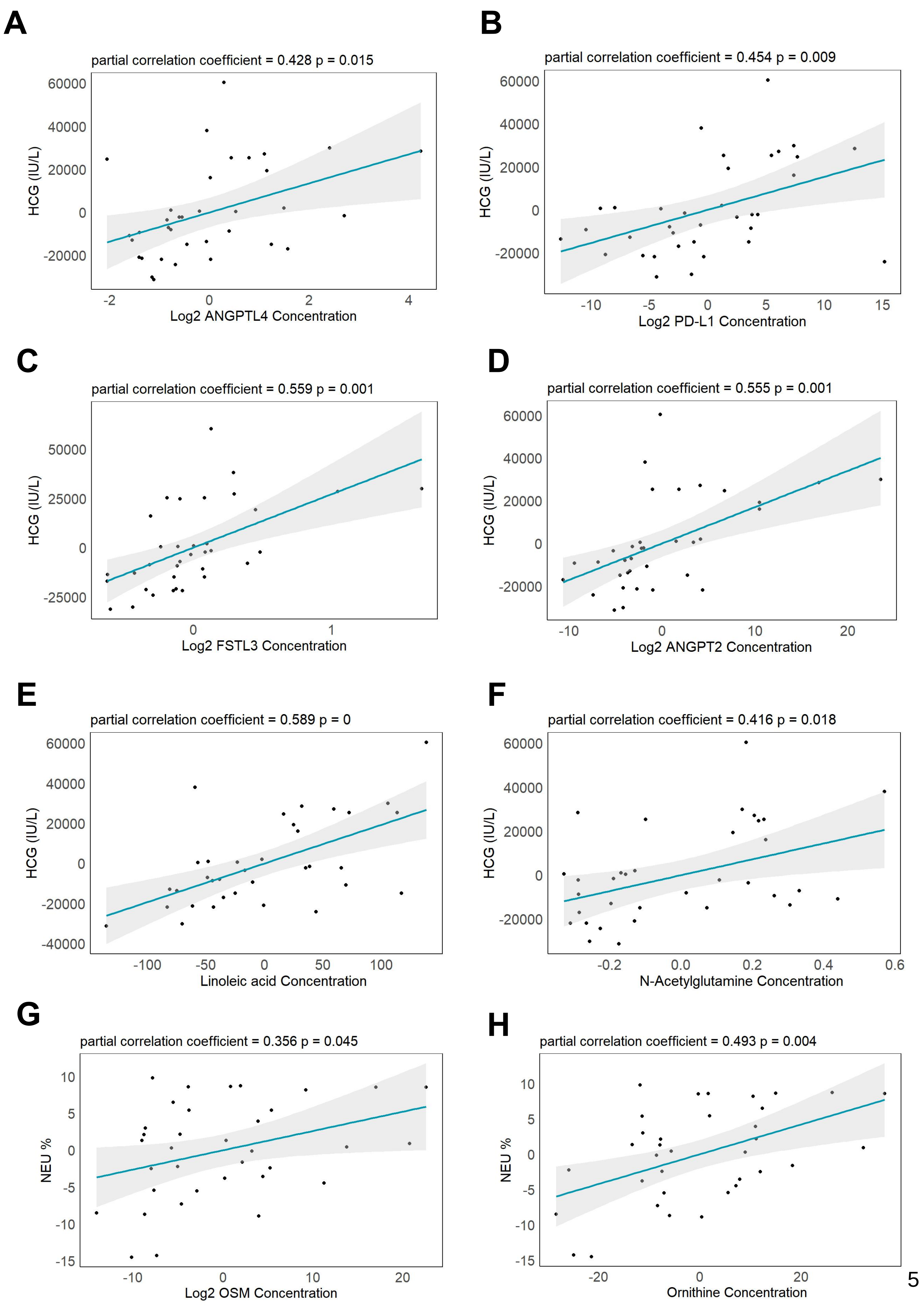

Supplementary Figure 4. Correlation between key proteins and metabolites and β-human chorionic gonadotropin and neutrophil proportion in patients with early pregnancy loss. Scatter plots of the correlation between serum levels of (**A**) β-HCG and ANGPTL4, (**B**) β-HCG and PD-L1, (**C**) β-HCG and FSTL3, (**D**) β-HCG and ANGPT2, (**E**) β-HCG and linoleic acid, (**F**) β-HCG and N-Acetylglutamine, (**G**) NEU% and OSM, and (**H**) NEU% and ornithine. Maternal age and gestational weeks were adjusted in the linear correlation. Abbreviation: Neutrophil (NEU), β-Human chorionic gonadotropin (β-HCG), Angiopoietin-related protein 4 (ANGPTL4), Follistatin-related protein 3 (FSTL3), Oncostatin M (OSM), Programmed cell death 1 ligand 1 (PD-L1), Angiopoietin-2 (ANGPT2).

Supplementary Figure 5

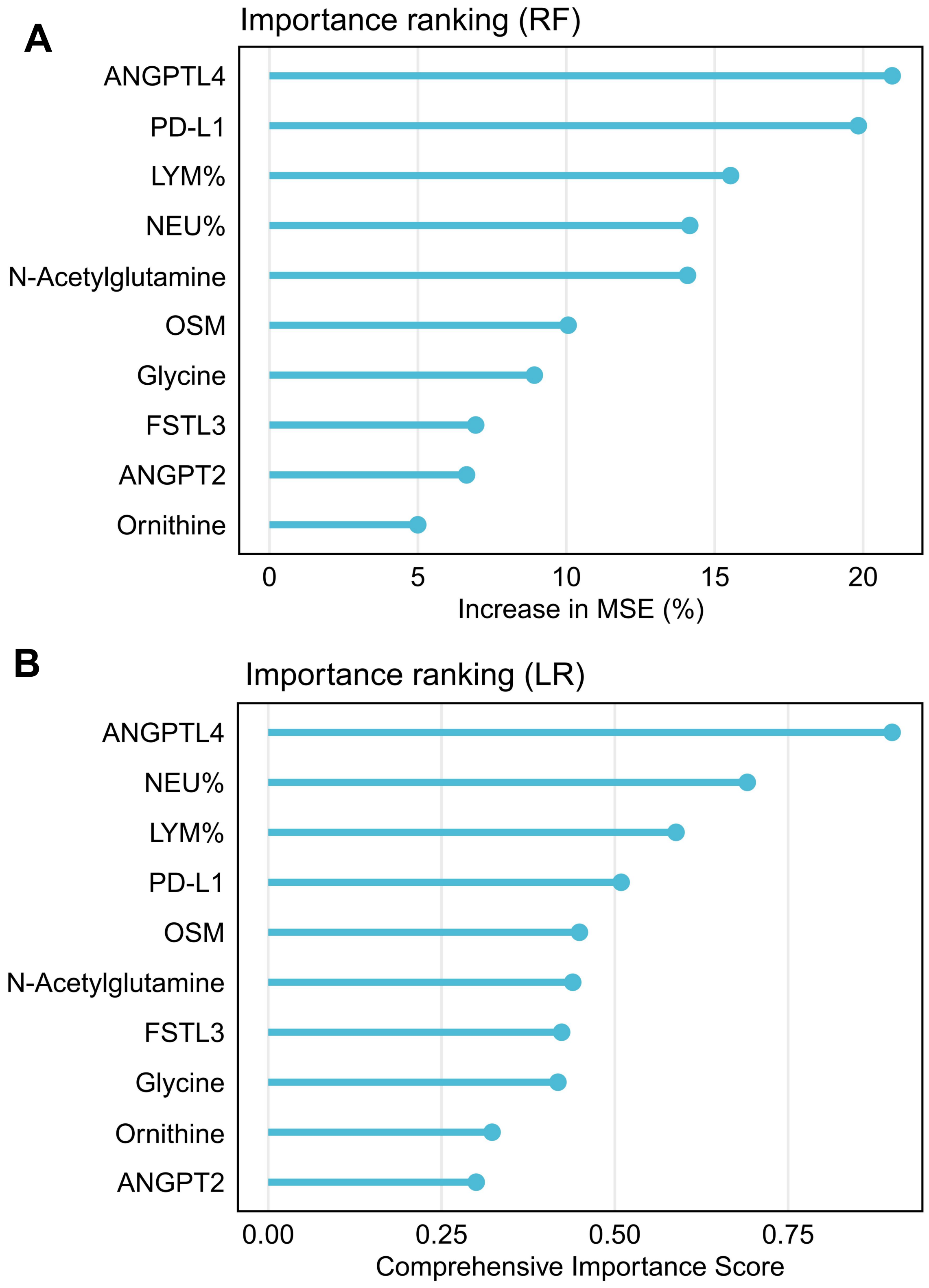

Supplementary Figure 5. Feature importance ranking in machine learning models. (**A**) The top 10 important features in the random forest algorithm determined by %IncMSE. (B) The top 10 important features in logistic regression ranked by comprehensive importance score (integrating coefficient, p-value, and performance drop after feature removal). Abbreviation: Random forest (RF), Percentage of increase in mean squared error (%IncMSE), Logistic regression (LR).

Supplementation Table 1. Protein analyzed in Olink Proteomics

Development Panel		Inflammation Panel		Metabolism Panel	
**Protein name**	**Uniport ID**	**Protein name**	**Uniport ID**	**Protein name**	**Uniport ID**
ADP-ribosyl cyclase/cyclic ADP-ribose hydrolase 2 (BST1)	Q10588	Adenosine Deaminase (ADA)	P00813	Adenosylhomocysteinase (AHCY)	P23526
ADP-sugar pyrophosphatase (NUDT5)	Q9UKK9	Artemin (ARTN)	Q5T4W7	Adhesion G protein-coupled receptor E2 (ADGRE2)	Q9UHX3
Aggrecan core protein (ACAN)	P16112	Axin-1 (AXIN1)	O15169	Adhesion G-protein coupled receptor G2 (ADGRG2)	Q8IZP9
Amyloid beta A4 protein (APP)	P05067	Beta-nerve growth factor (Beta-NGF)	P01138	Amyloid-like protein 1 (APLP1)	P51693
Angiopoietin-related protein 4 (ANGPTL4)	Q9BY76	Caspase-8 (CASP-8)	Q14790	Angiopoietin-2 (ANGPT2)	O15123
Arylsulfatase A (ARSA)	P15289	C-C motif chemokine 19 (CCL19)	Q99731	Angiopoietin-related protein 1 (ANGPTL1)	O95841
Basal cell adhesion molecule (BCAM)	P50895	C-C motif chemokine 20 (CCL20)	P78556	Angiopoietin-related protein 7 (ANGPTL7)	O43827
Beta-1,4-galactosyltransferase 1 (B4GALT1)	P15291	C-C motif chemokine 23 (CCL23)	P55773	Annexin A11 (ANXA11)	P50995
Beta-1,4-glucuronyltransferase 1 (B4GAT1)	O43505	C-C motif chemokine 25 (CCL25)	O15444	Annexin A4 (ANXA4)	P09525
Beta-glucuronidase (GUSB)	P08236	C-C motif chemokine 28 (CCL28)	Q9NRJ3	Appetite-regulating hormone (GHRL)	Q9UBU3
Beta-microseminoprotein (MSMB)	P08118	C-C motif chemokine 3 (CCL3)	P10147	Arginase-1 (ARG1)	P05089
Carbonic anhydrase 2 (CA2)	P00918	C-C motif chemokine 4 (CCL4)	P13236	Aromatic-L-amino-acid decarboxylase (DDC)	P20711
Carbonic anhydrase 6 (CA6)	P23280	CD40L receptor (CD40)	P25942	B-cell antigen receptor complex-associated protein beta chain (CD79B)	P40259
Cathepsin F (CTSF)	Q9UBX1	CUB domain-containing protein 1 (CDCP1)	Q9H5V8	Cadherin-2 (CDH2)	P19022
Cation-independent mannose-6-phosphate receptor (IGF2R)	P11717	C-X-C motif chemokine 1 (CXCL1)	P09341	Cadherin-related family member 5 (CDHR5)	Q9HBB8
C-C motif chemokine 21 (CCL21)	O00585	C-X-C motif chemokine 10 (CXCL10)	P02778	Calsyntenin-2 (CLSTN2)	Q9H4D0
CD109 antigen (CD109)	Q6YHK3	C-X-C motif chemokine 11 (CXCL11)	O14625	Carbonic anhydrase 13 (CA13)	Q8N1Q1
CD177 antigen (CD177)	Q8N6Q3	C-X-C motif chemokine 5 (CXCL5)	P42830	Catechol O-methyltransferase (COMT)	P21964
CD209 antigen (CD209)	Q9NNX6	C-X-C motif chemokine 6 (CXCL6)	P80162	Cathepsin O (CTSO)	P43234
CD97 antigen (CD97)	P48960	C-X-C motif chemokine 9	Q07325	CD2-associated protein (CD2AP)	Q9Y5K6

		(CXCL9)			
CD99 antigen-like protein 2 (CD99L2)	Q8TCZ2	Cystatin D (CST5)	P28325	Chordin-like protein 2 (CHRDL2)	Q6WN34
Cell adhesion molecule-related/down-regulated by oncogenes (CDON)	Q4KMG0	Delta and Notch-like epidermal growth factor-related receptor (DNER)	Q8NFT8	Clusterin-like protein 1 (CLUL1)	Q15846
CMRF35-like molecule 9 (CD300LG)	Q6UXG3	Eotaxin (CCL11)	P51671	Coiled-coil domain-containing protein 80 (CCDC80)	Q76M96
Cochlin (COCH)	O43405	Eukaryotic translation initiation factor 4E-binding protein 1 (4E-BP1)	Q13541	Crk-like protein (CRKL)	P46109
Collectin-12 (COLEC12)	Q5KU26	Fibroblast growth factor 19 (FGF-19)	O95750	C-type lectin domain family 5 member A (CLEC5A)	Q9NY25
Contactin-4 (CNTN4)	Q8IWV2	Fibroblast growth factor 21 (FGF-21)	Q9NSA1	CXADR-like membrane protein (CLMP)	Q9H6B4
Corticotropin-releasing factor-binding protein (CRHBP)	P24387	Fibroblast growth factor 23 (FGF-23)	Q9GZV9	Diablo homolog, mitochondrial (DIABLO)	Q9NR28
C-type lectin domain family 11 member A (CLEC11A)	Q9Y240	Fibroblast growth factor 5 (FGF-5)	P12034	Dihydropteridine reductase (QDPR)	P09417
C-type lectin domain family 14 member A (CLEC14A)	Q86T13	Fms-related tyrosine kinase 3 ligand (Flt3L)	P49771	Dipeptidyl peptidase 2 (DPP7)	Q9UHL4
Cystatin-M (CST6)	Q15828	Fractalkine (CX3CL1)	P78423	Disabled homolog 2 (DAB2)	P98082
Cysteine-rich motor neuron 1 protein (CRIM1)	Q9NZV1	Glial cell line-derived neurotrophic factor (GDNF)	P39905	DNA-(apurinic or apyrimidinic site) lyase (APEX1)	P27695
Cysteine-rich with EGF-like domain protein 2 (CRELD2)	Q6UXH1	Hepatocyte growth factor (HGF)	P14210	Ectonucleoside triphosphate diphosphohydrolase 5 (ENTPD5)	O75356
Cytosolic phospholipase A2 (PLA2G4A)	P47712	Interferon gamma (IFN-gamma)	P01579	Ectonucleotide pyrophosphatase/phosphodiesterase family member 7 (ENPP7)	Q6UWV6
Desmocollin-2 (DSC2)	Q02487	Interleukin-1 alpha (IL-1 alpha)	P01583	Eosinophil cationic protein (RNASE3)	P12724
Dickkopf-related protein 3 (DKK3)	Q9UBP4	Interleukin-10 (IL10)	P22301	Fc receptor-like protein 1 (FCRL1)	Q96LA6
Dystroglycan (DAG1)	Q14118	Interleukin-10 receptor subunit alpha (IL-10RA)	Q13651	Fructose-1,6-bisphosphatase 1 (FBP1)	P09467
Early activation antigen CD69 (CD69)	Q07108	Interleukin-10 receptor subunit beta (IL-10RB)	Q08334	Galanin peptides (GAL)	P22466
Ectonucleotide pyrophosphatase/phosphodiesterase family member 2 or Autotaxin (ENPP2)	Q13822	Interleukin-12 subunit beta (IL-12B)	P29460	Gamma-enolase (ENO2)	P09104

Endothelial cell-selective adhesion molecule (ESAM)	Q96AP7	Interleukin-13 (IL-13)	P35225	Glutaredoxin-1 (GLRX)	P35754
Fc receptor-like protein 5 (FCRL5)	Q96RD9	Interleukin-15 receptor subunit alpha (IL-15RA)	Q13261	GRB2-related adapter protein 2 (GRAP2)	O75791
Flavin reductase NADPH (BLVRB)	P30043	Interleukin-17A (IL-17A)	Q16552	Hepatoma-derived growth factor (HDGF)	P51858
Follistatin-related protein 3 (FSTL3)	O95633	Interleukin-17C (IL-17C)	Q9P0M4	Inactive tyrosine-protein kinase transmembrane receptor ROR1 (ROR1)	Q01973
Galactoside 3(4)-L-fucosyltransferase,Alpha-(1, 3)-fucosyltransferase 3/5 (FUT3/5)	P21217,Q11128	Interleukin-18 (IL-18)	Q14116	Insulin-like growth factor-binding protein-like 1 (IGFBPL1)	Q8WX77
Glycoprotein hormones alpha chain (CGA)	P01215	Interleukin-18 receptor 1 (IL-18R1)	Q13478	Integrin beta-7 (ITGB7)	P26010
Glycoprotein Xg (XG)	P55808	Interleukin-2 (IL-2)	P60568	Kallikrein-10 (KLK10)	O43240
Hepatitis A virus cellular receptor 2 (HAVCR2)	Q8TDQ0	Interleukin-2 receptor subunit beta (IL-2RB)	P14784	Kynurenine--oxoglutarate transaminase 1 (KYAT1)	Q16773
HLA class II histocompatibility antigen gamma chain (CD74)	P04233	Interleukin-20 (IL-20)	Q9NYY1	Large proline-rich protein BAG6 (BAG6)	P46379
Inactive serine protease PAMR1 (PAMR1)	Q6UXH9	Interleukin-20 receptor subunit alpha (IL-20RA)	Q9UHF4	Leucine-rich repeats and immunoglobulin-like domains protein 1 (LRIG1)	Q96JA1
Inhibin beta C chain (INHBC)	P55103	Interleukin-22 receptor subunit alpha-1 (IL-22 RA1)	Q8N6P7	Leukocyte immunoglobulin-like receptor subfamily A member 5 (LILRA5)	A6NI73
Integrin alpha-5 (ITGA5)	P08648	Interleukin-24 (IL-24)	Q13007	Low-density lipoprotein receptor-related protein 11 (LRP11)	Q86VZ4
Integrin beta-1 (ITGB1)	P05556	Interleukin-33 (IL-33)	O95760	Lysophosphatidic acid phosphatase type 6 (ACP6)	Q9NPH0
Interleukin-13 receptor subunit alpha-1 (IL13RA1)	P78552	Interleukin-4 (IL-4)	P05112	Meprin A subunit beta (MEP1B)	Q16820
Kunitz-type protease inhibitor 1 (SPINT1)	O43278	Interleukin-5 (IL5)	P05113	Meteorin-like protein (METRNL)	Q641Q3
Kunitz-type protease inhibitor 2 (SPINT2)	O43291	Interleukin-6 (IL6)	P05231	Multiple coagulation factor deficiency protein 2 (MCFD2)	Q8NI22
Lactadherin (MFGE8)	Q08431	Interleukin-7 (IL-7)	P13232	NAD kinase (NADK)	O95544
Laminin subunit alpha-4 (LAMA4)	Q16363	Interleukin-8 (IL-8)	P10145	Nectin-2 (NECTIN2)	Q92692
LDLR chaperone MESD (MESDC2)	Q14696	Latency-associated peptide transforming growth factor beta-1 (LAP TGF-beta-1)	P01137	Neural proliferation differentiation and control protein 1 (NPDC1)	Q9NQX5
Legumain (LGMN)	Q99538	Leukemia inhibitory factor (LIF)	P15018	Neuronal pentraxin receptor	O95502

				(NPTXR)	
Leukocyte-associated immunoglobulin-like receptor 1 (LAIR1)	Q6GTX8	Leukemia inhibitory factor receptor (LIF-R)	P42702	Nodal modulator 1 (NOMO1)	Q15155
Low affinity immunoglobulin epsilon Fc receptor (FCER2)	P06734	Macrophage colony-stimulating factor 1 (CSF-1)	P09603	N-terminal prohormone brain natriuretic peptide (NT-proBNP)	NA
Lymphocyte function-associated antigen 3 (CD58)	P19256	Matrix metalloproteinase-1 (MMP-1)	P03956	Paired immunoglobulin-like type 2 receptor beta (PILRB)	Q9UKJ0
Macrophage migration inhibitory factor (MIF)	P14174	Matrix metalloproteinase-10 (MMP-10)	P09238	Peptidyl-prolyl cis-trans isomerase FKBP4 (FKBP4)	Q02790
Matrilin-2 (MATN2)	O00339	Monocyte chemotactic protein 1 (MCP-1)	P13500	Phosphoprotein associated with glycosphingolipid-enriched microdomains 1 (PAG1)	Q9NWQ8
Myocilin (MYOC)	Q99972	Monocyte chemotactic protein 2 (MCP-2)	P80075	Pro-cathepsin H (CTSH)	P09668
Nidogen-2 (NID2)	Q14112	Monocyte chemotactic protein 3 (MCP-3)	P80098	Protein FAM3C (FAM3C)	Q92520
Osteomodulin (OMD)	Q99983	Monocyte chemotactic protein 4 (MCP-4)	Q99616	Protein phosphatase inhibitor 2 (PPP1R2)	P41236
Paired immunoglobulin-like type 2 receptor alpha (PILRA)	Q9UKJ1	Natural killer cell receptor 2B4 (CD244)	Q9BZW8	Protein S100-P (S100P)	P25815
Peptidyl-prolyl cis-trans isomerase B (PPIB)	P23284	Neurotrophin-3 (NT-3)	P20783	Regenerating islet-derived protein 4 (REG4)	Q9BYZ8
Phosphatidylethanolamine-binding protein 1 (PEBP1)	P30086	Neurturin (NRTN)	Q99748	Reticulon-4 receptor (RTN4R)	Q9BZR6
Platelet endothelial aggregation receptor 1 (PEAR1)	Q5VY43	Oncostatin-M (OSM)	P13725	Retinal dehydrogenase 1 (ALDH1A1)	P00352
Platelet-derived growth factor receptor beta (PDGFRB)	P09619	Osteoprotegerin (OPG)	O00300	Ribosyldihydronicotinamide dehydrogenase (quinone) (NQO2)	P16083
Protein deglycase DJ-1 (PARK7)	Q99497	Programmed cell death 1 ligand 1 (PD-L1)	Q9NZQ7	Scavenger receptor cysteine-rich domain-containing group B protein (SSC4D)	Q8WTU2
Protein disulfide-isomerase (P4HB)	P07237	Protein S100-A12 (EN-RAGE)	P80511	Sclerostin (SOST)	Q9BQB4
Protein NOV homolog (NOV)	P48745	Signaling lymphocytic activation molecule (SLAMF1)	Q13291	Semaphorin-3F (SEMA3F)	Q13275
Receptor-type tyrosine-protein phosphatase F (PTPRF)	P10586	SIR2-like protein 2 (SIRT2)	Q8IXJ6	Serpin B6 (SERPINB6)	P35237

Roundabout homolog 1 (ROBO1)	Q9Y6N7	STAM-binding protein (STAMPB)	O95630	Serpin B8 (SERPINB8)	P50452
Scavenger receptor class F member 1 (SCARF1)	Q14162	Stem cell factor (SCF)	P21583	Sialic acid-binding Ig-like lectin 7 (SIGLEC7)	Q9Y286
Secretoglobin family 3A member 1 (SCGB3A1)	Q96QR1	Sulfotransferase 1A1 (ST1A1)	P50225	Sialomucin core protein 24 (CD164)	Q04900
Semaphorin-7A (SEMA7A)	O75326	T cell surface glycoprotein CD6 isoform (CD6)	P30203	Soluble calcium-activated nucleotidase 1 (CANT1)	Q8WVQ1
Serine protease HTRA2, mitochondrial (HTRA2)	O43464	T-cell surface glycoprotein CD5 (CD5)	P06127	Sulfatase-modifying factor 2 (SUMF2)	Q8NBJ7
Serine protease inhibitor Kazal-type 1 (SPINK1)	P00995	T-cell surface glycoprotein CD8 alpha chain (CD8A)	P01732	Synaptosomal-associated protein 23 (SNAP23)	O00161
Serine protease inhibitor Kazal-type 5 (SPINK5)	Q9NQ38	Thymic stromal lymphopoietin (TSLP)	Q969D9	Syndecan-4 (SDC4)	P31431
Signal-regulatory protein beta-1 (SIRPB1)	O00241	TNF-beta (TNFB)	P01374	T-cell surface glycoprotein CD1c (CD1C)	P29017
Stress-induced-phosphoprotein 1 (STIP1)	P31948	TNF-related activation-induced cytokine (TRANCE)	O14788	Thimet oligopeptidase (THOP1)	P52888
Synaptosomal-associated protein 29 (SNAP29)	O95721	TNF-related apoptosis-inducing ligand (TRAIL)	P50591	Thioredoxin domain-containing protein 5 (TXNDC5)	Q8NBS9
Thymosin beta-10 (TMSB10)	P63313	Transforming growth factor alpha (TGF-alpha)	P01135	Thymidine phosphorylase (TYMP)	P19971
Tissue alpha-L-fucosidase (FUCA1)	P04066	Tumor necrosis factor (Ligand) superfamily, member 12 (TWEAK)	O43508	Thyrotropin subunit beta (TSHB)	P01222
Tripeptidyl-peptidase 1 (TPP1)	O14773	Tumor necrosis factor (TNF)	P01375	Trefoil factor 2 (TFF2)	Q03403
Tumor necrosis factor receptor superfamily member 19L (RELT)	Q969Z4	Tumor necrosis factor ligand superfamily member 14 (TNFSF14)	O43557	Tubulointerstitial nephritis antigen-like (TINAGL1)	Q9GZM7
Tyrosine-protein phosphatase non-receptor type 6 (PTPN6)	P29350	Tumor necrosis factor receptor superfamily member 9 (TNFRSF9)	Q07011	Tyrosine-protein kinase receptor TYRO3 (TYRO3)	Q06418
WAP, Kazal, immunoglobulin, Kunitz and NTR domain-containing protein 2 (WFIKKN2)	Q8TEU8	Urokinase-type plasminogen activator (uPA)	P00749	Ubiquitin carboxyl-terminal hydrolase 8 (USP8)	P40818
V-set and immunoglobulin domain-containing protein 4 (VSIG4)	Q9Y279	Vascular endothelial growth factor A (VEGF-A)	P15692	Versican core protein (VCAN)	P13611

Supplementation Table 2. Metabolites quantified by UPLCMS/MS method

Name (Abbreviation)		
Lysine	N-Acetylneuraminic acid	Myristoleic acid
Histidine	Acetic acid	9E-tetradecenoic acid
Arginine	Valine	Ricinoleic acid
Ornithine	Lactulose	Ricinelaidic acid
Glutamine	Maltose	Deoxycholic acid (DCA)
Glutamic acid	3-Hydroxybutyric acid	Myristic acid
Sarcosine	Methionine	9-Pentadecenoic acid
beta-Alanine	alpha-Hydroxyisobutyric acid	Pentadecanoic acid
Alanine	2-Hydroxybutyric acid	Palmitoleic acid
Dimethylglycine	3-Hydroxyisovaleric acid	Palmitelaidic acid
Gamma-aminobutyric acid (GABA)	Isoleucine	10Z-Heptadecenoic acid
Serine	Leucine	10-Trans-Heptadecenoic acid
Threonine	Xylose	alpha-Linolenic acid
Creatine	Ribulose|Xylulose	gamma-Linolenic acid
Hydroxypropionic acid	Fructose	Linoleic acid
Glycylproline	N-Acetylglucosamine	Linoelaidic acid
Homocitrulline	Propionic acid	(10Z,13Z)-Nonadecadienoic acid
Adenosine monophosphate (AMP)	Norleucine	Eicosapentaenoic acid (EPA)
Lactic acid	Homovanillic acid	Arachidonic acid
Methylcysteine	3-(3-Hydroxyphenyl)-3-hydroxypropanoic acid (HPHPA)	Dihomo-gamma-linolenic acid
2-Phenylglycine	p-Hydroxyphenylacetic acid	Docosahexaenoic acid (DHA)
Tyrosine	3-Hydroxyanthranilic acid	Docosapentaenoic acid (DPA)
Asparagine	Tryptophan	Docosapentaenoic acid 22n_6 (DPAn-6)
Phenylalanine	Butyric acid	Adrenic acid
Hydroxyphenyllactic acid	Isobutyric acid	Succinic acid
Glycylleucine	Malonic acid	Citramalic acid
Kynurenine	Phenylacetylglutamine	Oleic acid
Aspartic acid	Isovaleric acid	10Z-Nonadecenoic acid
Aminoadipic acid	Valeric acid	3-Hydroxyphenylacetic acid
2-Butenoic acid	Phenyllactic acid	Carnitine
2-Hydroxy-3-methylbutyric acid	Citraconic acid	Acetylcarnitine
Hippuric acid	Methylglutaric acid	Propionylcarnitine
N-Acetyl-L-alanine	Phthalic acid	Malonylcarnitine
Malic acid	2-Methylpentanoic acid	Butyrylcarnitine
N-Acetylaspartic acid	Isocaproic acid	Isobutyrylcarnitine
N-Acetytyrosine	Taurocholic acid (TCA)	Valerylcarnitine
Ethylmethylacetic acid	Citric acid	2-Methylbutyroylcarnitine
2-Methy-4-pentenoic acid	Isocitric acid	Isovalerylcarnitine
2-Hydroxycaproic acid	3-Phenylbutyric acid	3-Hydroxylisovalerylcarnitine
Glutaric acid	2-Methylhexanoic acid	Glutarylcarnitine
trans-Aconitic acid	4-Methylhexanoic acid	Hexanoylcarnitine
N-Acetyltryptophan	Heptanoic acid	Adipoylcarnitine
Hydrocinnamic acid	Taurochenodeoxycholate (TCDCA)	Octanoylcarnitine
Azelaic acid	7-dehydrocholic acid (7-DHCA)	Decanoylcarnitine
Sebacic acid	3-dehydrocholic acid (3-DHCA)	Dodecanoylcarnitine

alpha-Ketoisovaleric acid	Oxoglutaric acid	Tetradecanoylcarnitine
Ketoleucine	Glycoursodeoxycholic acid (GUDCA)	Palmitoylcarnitine
3-Methyl-2-oxopentanoic acid	Glycohyodeoxycholate (GHDCA)	Oleylcarnitine
Phenylpyruvic acid	Octanoic acid	Linoleylcarnitine
Methylmalonic acid	Ursodeoxycholic acid (UDCA)	Stearylcarnitine
2-Hydroxyglutaric acid	Hyocholic acid (HCA)	Glucose 6-phosphate
Glycine	Cholic acid (CA)	Glycolithocholic acid 3-sulfate (GLCA-3S)
Citrulline	Oxoadipic acid	Methylmalonylcarnitine
Gluconolactone	Glycocholic acid (GCA)	S-Adenosylhomocysteine (SAH)
Glycolic acid	Decanoic acid	7-ketodeoxycholic acid (7-KetoLCA)
alpha-Aminobutyric acid	Undecylenic acid	β-Ursodeoxycholic Acid (βUDCA)
Glyceric acid	Chenodeoxycholic acid (CDCA)	12-ketolithocholic acid (12-KetoLCA)
Proline	Chenodeoxycholic acid glycine conjugate	N-Acetylhistidine
Acetylglycine	glycodeoxycholic acid (GDCA)	2,2-Dimethylsuccinic acid
Pipecolic acid	Undecanoic acid	Indolelactic acid
Erythronic acid	5Z-Dodecenoic acid	1-Methylhistidine
N-Acetylserine	Dodecanoic acid	4-Hydroxyproline
N-Acetylglutamine	12-Tridecenoic acid	gamma-Glutamylalanine
Quinic acid	Tridecanoic acid	Glycodeoxycholic acid 3-sulfate (GDCA-3S)
Chenodeoxycholic acid glycine conjugate 3 sulfate （GCDCA-3S)	Imidazolepropionic acid	

Supplementation Table 3 . Performance of the models in final random forest model determination

Model component	AUC	95% CI	Sensitivity	Specificity	Threshold	PPV	NPV	*p*-value
ANGPTL4+NEU+LYM+OSM+N-Acetylglutamine+FSTL3+Glycine+WBC+PD-L1+ANGPT2	0.994	0.976-1.000	0.923	1.000	0.664	1.000	0.923	0.008
ANGPTL4+NEU+LYM+OSM+N-Acetylglutamine+FSTL3+Glycine+WBC+PD-L1	1.000	1.000-1.000	1.000	1.000	0.609	1.000	1.000	0.007
ANGPTL4+NEU+LYM+OSM+N-Acetylglutamine+FSTL3+PD-L1+WBC	0.981	0.942-1.000	0.846	1.000	0.685	1.000	0.857	0.010
ANGPTL4+NEU+LYM+OSM+N-Acetylglutamine+FSTL3+PD-L1	1.000	1.000-1.000	1.000	1.000	0.625	1.000	1.000	0.007
ANGPTL4+NEU+LYM+OSM+N-Acetylglutamine+PD-L1	1.000	1.000-1.000	1.000	1.000	0.621	1.000	1.000	0.007
ANGPTL4+NEU+LYM+PD-L1+N-Acetylglutamine	0.994	0.976-1.000	0.923	1.000	0.721	1.000	0.923	0.008
ANGPTL4+NEU+LYM+PD-L1	0.944	0.835-1.000	0.833	1.000	0.548	1.000	0.818	0.024
ANGPTL4+NEU+LYM	0.897	0.777-1.000	0.846	0.833	0.730	0.846	0.833	0.059

Maternal age and gestational weeks were adjusted using the analysis of covariance (ANCOVA) method. The difference of ROC curves between biomarkers or models was estimated by Delong test. *P*-value implies the significance of the detective difference between the biomarker or model and HCG.

Abbreviation: Area under receiver operating characteristic curve (AUC), Positive predictive value (PPV), Negative predictive value (NPV),Angiopoietin-related protein 4 (ANGPTL4), Oncostatin M (OSM), Follistatin-related protein 3 (FSTL3), Soluble programmed cell death-1 ligand (PD-L1), Angiopoietin-2 (ANGPT2), White blood cell count (WBC), Neutrophil proportion (NEU), Lymphocyte proportion (LYM), Random forest (RF).

Supplementation Table 4 . Performance of the features in final logistic regression model determination

Feature	Standardized coefficient	*p*-value	Performance drop	Final score
ANGPTL4	405.611	0.996	0.001	0.900
NEU	221.588	0.998	0.045	0.691
LYM	197.907	0.998	0.001	0.588
PD-L1	40.869	0.999	0.045	0.509
OSM	51.279	1.000	0.045	0.449
N-Acetylglutamine	317.629	0.997	-0.045	0.439
FSTL3	29.582	1.000	0.045	0.423
Glycine	270.923	0.997	-0.136	0.418
Ornithine	210.540	0.998	-0.045	0.323
ANGPT2	14.540	1.000	0.001	0.300

The top-10 important variables in random forest model were used to build the initial logistic regression model. Final score was evaluated by 30% coefficient impact, 30% *p*-value impact (caluculated by Delong test), and 40% impact on overall performance drop after removing this feature. Maternal age and gestational weeks were adjusted using the analysis of covariance (ANCOVA) method.

Abbreviation: Angiopoietin-related protein 4 (ANGPTL4), Oncostatin M (OSM), Follistatin-related protein 3 (FSTL3), Soluble programmed cell death-1 ligand (PD-L1), Angiopoietin-2 (ANGPT2), Neutrophil proportion (NEU), Lymphocyte proportion (LYM).

Supplementary Table 5 .

Solo performance of key biomarkers in differentiating early pregnancy loss from healthy pregnancies

Biomarker	AUC	95% CI	Sensitivity	Specificity	Threshold	PPV	NPV	*p*-value
HCG	0.556	0.253-0.858	1.000	0.444	0.287	0.705	1.000	NA
ANGPT2	0.564	0.318-0.810	0.846	0.417	0.524	0.611	0.714	0.649
Ornithine	0.667	0.408-0.925	0.833	0.556	0.393	0.714	0.714	0.389
FSTL3	0.731	0.523-0.938	0.846	0.667	0.531	0.733	0.800	0.384
Glycine	0.750	0.548-0.952	0.846	0.667	0.406	0.733	0.800	0.371
OSM	0.782	0.600-0.964	0.692	0.750	0.577	0.750	0.692	0.265
N-Acetylglutamine	0.859	0.685-1.000	0.846	0.917	0.526	0.917	0.846	0.115

Maternal age and gestational weeks were adjusted using the analysis of covariance (ANCOVA) method. The difference of ROC curves between biomarkers or models was estimated by Delong test. *P*-value implies the significance of the detective difference between the biomarker or model and HCG

Abbreviation: Area under receiver operating characteristic curve (AUC), Positive predictive value (PPV), Negative predictive value (NPV), Oncostatin M (OSM), Follistatin-related protein 3 (FSTL3), Angiopoietin-2 (ANGPT2), Human chorionic gonadotropin (HCG).
