# Supplementary material for: Blood-based biomarker discovery for early pregnancy loss using integrative multi-omics strategies
Source: eBioMedicine. 2026 Apr 13;127:106253. doi: 10.1016/j.ebiom.2026.106253 (PMC13092690; doi:10.1016/j.ebiom.2026.106253)
Supplement: Reagent Validation [file mmc2.pdf]

Reagent Validation

Summary of ELISA kit performance

ELISA kit	Supplier cat. no	Sensitivity	Limit of detection	Inter-assay coefficient of variation	Intra-assay coefficient of variation
Human ANGPTL4	R&D, USA, DY3485	1.25-80 ng/mL	NA	0.27%	9.30%
Human PD-L1	R&D, USA, DB7H10	15.6-1000 pg/mL	25.81 pg/mL	1.01%	0.53%
